# Anti-BCMA CAR T-cell therapy CT103A in relapsed or refractory AQP4-IgG seropositive neuromyelitis optica spectrum disorders: phase 1 trial interim results

**DOI:** 10.1038/s41392-022-01278-3

**Published:** 2023-01-04

**Authors:** Chuan Qin, Dai-Shi Tian, Luo-Qi Zhou, Ke Shang, Liang Huang, Ming-Hao Dong, Yun-Fan You, Jun Xiao, Ying Xiong, Wen Wang, Hao Pang, Jing-Jing Guo, Song-Bai Cai, Di Wang, Chun-Rui Li, Min Zhang, Bi-Tao Bu, Wei Wang

**Affiliations:** 1 Department of Neurology, Tongji Hospital, Tongji Medical College, Huazhong University of Science and Technology, 430030 Wuhan, China; 2 Hubei Key Laboratory of Neural Injury and Functional Reconstruction, Huazhong University of Science and Technology, 430030 Wuhan, China; 3 Department of Hematology, Tongji Hospital, Tongji Medical College, Huazhong University of Science and Technology, 430030 Wuhan, China; 4 Department of Radiology, Tongji Hospital, Tongji Medical College, Huazhong University of Science and Technology, 430030 Wuhan, China; 5 Nanjing IASO Biotherapeutics Ltd, Nanjing, China

**Keywords:** Neurological disorders, Clinical trials

## Abstract

Chimeric antigen receptor (CAR) T-cell therapy that targets B-cell maturation antigen (BCMA) have great potentials in autoimmune diseases and could be novel therapeutics for relapsed/refractory neuromyelitis optica spectrum disorder (NMOSD). To evaluate the safety and efficacy of the CT103A, a self-developed BCMA-targeting CAR construct against BCMA, in patients with AQP4-IgG seropositive NMOSD, an ongoing, investigator-initiated, open-label, single-arm, phase 1 clinical trial is conducted at our center. In total, 12 patients were administered with a CAR-BCMA infusion. Ten of the 12 patients dosed were women (83.3%), with a median age of 49.5 years (range, 30–67). were The most common events of grade 3 or higher were hematologic toxic effects. Seven patients (58%) developed infections, but no grade 4 infections occurred. Cytokine release syndrome was reported in all patients with only events of grade 1 or 2 observed. During the follow-up of a median 5.5 months, 11 patients had no relapse; all patients generally reported improvement in disabilities and quality-of-life outcomes; 11 patients’ AQP-4 antibodies in serum showed a downward trend by the cutoff date. CAR T-cell expansion was associated with responses, and persisted more than 6 months post-infusion in 17% of the patients. In summary, CAR T-cell therapy shows a manageable safety profile and therapeutic potentials for patients with relapsed/refractory AQP4-IgG seropositive NMOSD. Another expansion phase is currently underway to determine the safety and efficacy of CAR T-BCMA infusion in patients with other neuro-inflammatory diseases.

## Introduction

Neuromyelitis optica spectrum disorder (NMOSD) is an autoimmune, inflammatory disorder of the central nervous system (CNS),^1^ with a relapsing disease course and serious sequelae. The discovery of the anti-aquaporin 4 (AQP4) autoantibodies allowed understanding of its pathophysiology and precision targeted therapy.^2^ Polyclonal immunoglobulin G (IgG) produced from plasma cells binds with AQP4 on the optic nerve and spinal cord, leading to multiple pathological outcomes.^2^

Oral immunosuppressants are used for NMOSD relapse prevention in last decade; however, 25–60% of patients continue to have recurrent attacks.^3^ Recently, several monoclonal antibodies targeting different pathophysiological processes have been proved effective for NMOSD treatment.^2^ Among them, monoclonal antibodies against B lymphocytes, were recognized to be therapeutically powerful against NMOSD.^2^ However, therapies targeting the B-lymphocyte antigen CD20 did not correlate with a decrease in serum levels of AQP4-IgG and were ineffective in some patients,^2,4–7^ possibly due to the incomplete depletion of AQP4-IgG. A novel therapy targeting AQP4-IgG-producing cells might be more specific and effective for AQP4-IgG seropositive NMOSD. In addition, physical barriers in vivo, such as blood brain barrier, can drastically reduce the effective biodistribution of monoclonal antibodies to the targeted cells in CNS.^8^

Chimeric antigen receptor (CAR) T-cell therapy becomes an emerging treatment option for long-term disease control in several hematologic cancers,^9–11^ and the therapeutic potential has been extent to treat autoimmune diseases such as pemphigus vulgaris and systemic lupus erythematosus (SLE).^12,13^ In contrast with monoclonal antibodies, CAR-engineered T cells, have the multiple advantages of tissue biodistribution property of cellular vehicles and the self-amplification property, while preserving the same antigen specificity.^14^ B-cell maturation antigen (BCMA) is a member of the tumor necrosis factor superfamily of proteins, which is primarily expressed on plasma cells and some mature B cells. In our recent study, a self-developed BCMA-targeting CAR construct (CT103A) showed a powerful clinical efficacy after single-dose administration in multiple myeloma (MM).^10,15^ However, CAR-BCMA T-cell therapy has never been reported in treating autoimmune diseases. Based on the key role of the production of pathogenic AQP4-IgG in NMOSD and resident plasma cells in CNS, we speculated that this anti-BCMA CAR T-cell therapy might have powerful potentials in plasma cell depleting and clinical efficacy in patients with AQP4-IgG seropositive NMOSD.

## Results

### Patients

**Fig. 1:**
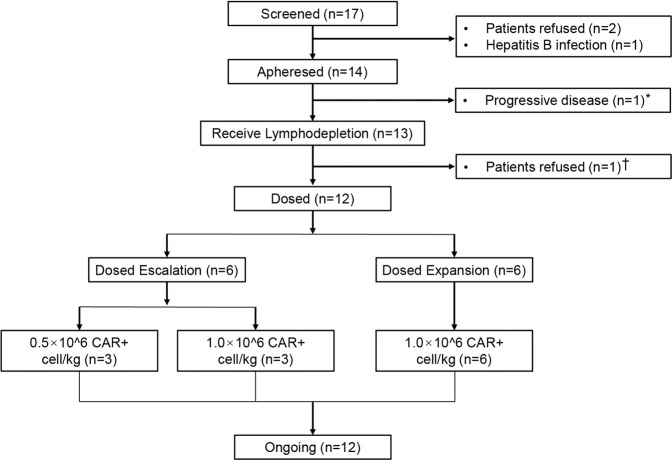
CONSORT diagram. *One patient suffered a severe NMOSD attack while her manufacturing failed. She refused another apheresis and discontinued before lymphodepletion. ^†^One patient underwent leukapheresis but discontinued before initial CT103A infusion for personal reason

**Fig. 2:**
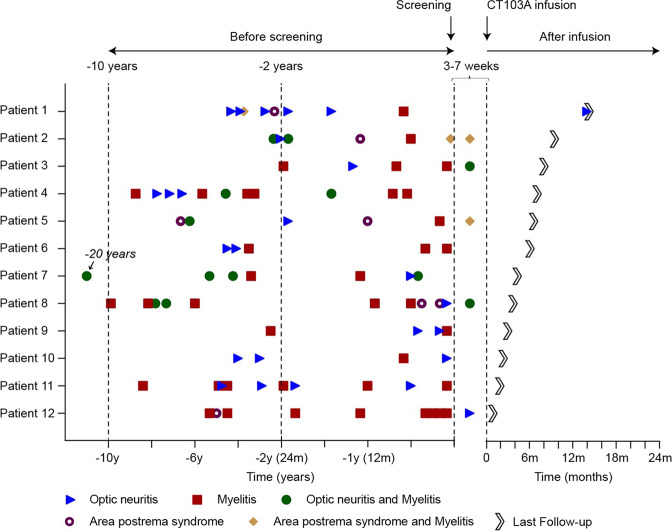
Attack frequency before, during, and after CAR T therapy

From September 22, 2020, to December 24, 2021, a total of 17 patients were screened, and 14 patients were apheresed. CT103A was successfully manufactured in 13 patients; 1 was not dosed for personal reason (Fig. 1). Ten of the 12 patients were women (83.3%), with a median age of 49.5 years (range, 30–67). Before enrollment, all 12 patients received oral corticosteroids and immunosuppressant drugs for attack prevention (Table 1 and Supplementary Table S1). Their attacks continued despite several immunosuppressant therapies (Fig. 2). In the year preceding enrollment, 30 attacks were reported in all patients (median 2 per patient, range 1 to 5). Five attacks in separate patients occurred between the screening visit and the CT103A infusion.

**Table 1:** Demographic and clinical characteristics of the patients at baseline

Characteristic	0.5 × 10^6^ CAR^+^ cell/kg (*N* = 3)	1.0 × 10^6^ CAR^+^ cell/kg (*N* = 9)	All patients (*N* = 12)
Female sex—no. (%)	2 (67)	8 (89)	10 (83)
Age, years—median (range)			
At initial clinical presentation	55.0 (27.0–57.0)	42.0 (22.0–63.0)	42.5 (22.0–63.0)
At first receipt of trial agent	57.0 (31.0–60.0)	47.0 (30.0–67.0)	49.5 (30.0–67.0)
Disease duration, years—median (range)	2.0 (2.0–4.0)	6.0 (2.0–20.0)	4.5 (2.0–20.0)
Annualized relapse rate during—median (range)			
Previous 24 months	2.0 (1.5–2.0)	1.5 (1.0–3.0)	1.5 (1.0–3.0)
Previous 12 months	2.0 (1.0–2.0)	2.0 (2.0–5.0)	2.0 (1.0–5.0)
Type of relapse during previous 24 months—no. (%)			
Optic neuritis	3 (27)	9 (26)	12 (27)
Acute myelitis	5 (45)	19 (56)	24 (53)
Area postrema syndrome	1 (9)	3 (9)	4 (9)
Time since last relapse (months)—median (range)	1.0 (1.0–6.0)	1.0 (1.0–7.0)	1.0 (1.0–7.0)
Previous treatment—no. (%)			
Previous rescue treatment			
Any therapy	3 (100)	9 (100)	12 (100)
Intravenous corticosteroids	3 (100)	9 (100)	12 (100)
Plasmapheresis	1 (33)	4 (44)	5 (42)
Intravenous immunoglobulin	3 (100)	6 (67)	9 (75)
Previous maintenance therapy			
Any previous maintenance therapy	3 (100)	9 (100)	12 (100)
Oral corticosteroids	3 (100)	9 (100)	12 (100)
Azathioprine	1 (33)	5 (56)	6 (50)
Mycophenolate mofetil	1 (33)	5 (56)	6 (50)
Tacrolimus	3 (100)	5 (56)	8 (67)
Methotrexate	0 (0)	1 (11)	1 (8)
Rituximab	1 (33)	1 (11)	2 (17)
Concomitant autoimmune diseases—no. (%)			
Any	3 (100)	3 (33)	6 (50)
Sjögren’s syndrome	0 (0)	3 (33)	3 (25)
Rheumatoid arthritis	0 (0)	1 (11)	1 (8)
Autoimmune thyroiditis	2 (67)	1 (11)	3 (25)
Acute urticaria	1 (33)	0 (0)	1 (8)
EDSS score—median (range)	7.0 (4.5–7.0)	4.5 (2.5–7.5)	4.5 (2.5–7.5)

Scores on the Expanded Disability Status Scale (EDSS) range from 0 (normal neurologic examination) to 10 (death)

### Safety

All patients had adverse events (AEs) of grade 3 or higher (Table 2, AEs categorized by investigators as being related to a trial regimen are described in Supplementary Table S2). Hematological toxic effects were the most common events of grade 3 or higher, including leukopenia (100%), neutropenia (100%), anemia (50%), and thrombocytopenia (25%), most of which was resolved within 4 weeks (Supplementary Fig. S1). Infection developed in 7(58%) patients. No grade 4 infections were observed. Treatment-related serious adverse events (SAEs) recorded were cytomegalovirus infection (25%), coagulation disorder (8%), and pneumonia (8%) (Supplementary Table S3).

**Table 2:** Adverse events, cytokine release syndrome, and neurologic toxic effects related to trial regimen

Variable	All patients (*N* = 12)	
	Any-grade	Grade 3 or higher
	Number of patients (%*)*	
Adverse event		
Any	12 (100)	12 (100)
Hematologic	12 (100)	12 (100)
Leukopenia	12 (100)	12 (100)
Neutropenia	12 (100)	12 (100)
Anemia	10 (83)	6 (50)
Thrombocytopenia	3 (25)	3 (25)
Lymphocytopenia	12 (100)	12 (100)
Platelet count increased	1 (8)	0 (0)
Leukocytosis	1 (8)	0 (0)
Neutrophilia	1 (8)	0 (0)
Gastrointestinal	4 (33)	1 (8)
Nausea and vomiting	3 (25)	0 (0)
Diarrhea	4 (33)	1 (8)
Infectious	7 (58)	7 (58)
Upper respiratory infection	1 (8)	1 (8)
CMV infection	5 (42)	5 (42)
Urinary infection	4 (33)	3 (25)
Oral herpes	1 (8)	0 (0)
Pneumonia	1 (8)	1 (8)
EBV infection	1 (8)	0 (0)
BKV infection	1 (8)	0 (0)
Other		
Flu like symptoms	5 (42)	0 (0)
Pyrexia	12 (100)	0 (0)
Hypotension	1 (8)	0 (0)
Expectoration	1 (8)	0 (0)
AST increased	3 (25)	0 (0)
ALT increased	2 (17)	1 (8)
Blood LDH increased	1 (8)	0 (0)
APTT prolonged	1 (8)	0 (0)
Coagulation disorder	1 (8)	1 (8)
Hematuria	2 (17)	0 (0)
Hypocalcemia	1 (8)	0 (0)
Hypokalemia	1 (8)	1 (8)
Blood fibrinogen decreased	1 (8)	0 (0)
NT-proBNP increased	2 (17)	0 (0)
Myocardial strain	1 (8)	0 (0)
Hypogammaglobulinemia	11 (92)	0 (0)
Cytokine release syndrome	12 (100)	0 (0)
Neurologic toxic effect	0 (0)	0 (0)

*CMV* cytomegalovirus infection, *BKV* BK virus, *EBV* Epstein–Barr virus, *APTT* activated partial thromboplastin time, *NT-proBNP* N-terminal pro-brain natriuretic peptides, *AST* aspartate aminotransferase, *ALT* alanine aminotransferase, *LDH* lactate dehydrogenase

All patients had grade 1 or 2 cytokine release syndrome (CRS); there were no cases of grade 3 or higher CRS (Tables 2 and 3). Three patients received glucocorticoids, and none required tocilizumab therapy. No neurologic toxic effect including immune effector cell-associated neurotoxicity syndrome (ICANS), dose-limiting-toxicity, and new-onset parkinsonism were observed, suggesting that a dose of ≤1.0 × 10^6^ CAR T cells/kg was well tolerated in patients with NMOSD.

**Table 3:** Characteristics and management of cytokine release syndrome

Parameter	Total (*n* = 12)
Patients with a CRS event—no. (%)	
Any-grade	12 (100)
Grade ≥3	0 (0)
Median (min–max) time to onset, days	
Any-grade	7.0 (1.0–15.0)
Grade ≥3	-
Median (min–max) duration, days	
Any-grade	3.5 (1.0–8.0)
Grade ≥3	-
Tocilizumab use—no. (%)^a^	0 (0)
Corticosteroid use—no. (%)^b^	3 (25)

*CRS* cytokine release syndrome

^a^No one was in need of tocilizumab at the treating physician’s discretion based on protocol-specified toxicity management guidelines

^b^Steroid doses administered for treatment of CRS included single dose of methylprednisolone 40 mg for Patient 2 and 9 within 10 days post infusion, and two doses of methylprednisolone 40 mg on D6 and D13 for Patient 7

### Expansion and persistence

**Fig. 3:**
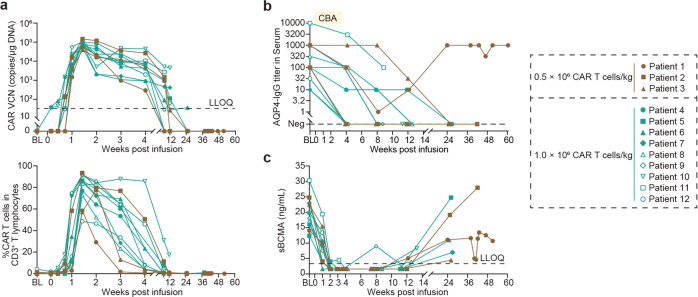
CT103A expansion and persistence, kinetic changes of serum AQP4-IgG and BCMA post-infusion. **a** Kinetic changes of CAR transgene detected by ddPCR and CAR T-cell percentage in CD3^+^ T lymphocytes detected by flowcytometry post infusion. LLOQ denotes lower limit of quantification (32 copies/μg DNA). **b**, **c** Kinetic changes of serum AQP4-IgG detected by cell-based assay (CBA) and BCMA post-infusion. LLOQ denotes lower limit of quantification (3.2 ng/mL). Levels of sBCMA lower than LLOQ were shown as 1/2 LLOQ

Following CT103A infusion, the CAR-positive cells proliferate and undergo rapid multi-log expansion and then gradually declined (Fig. 3a). The median time of maximal expansion occurred 10 days after infusion for both dose levels. Peak level and exposure were slightly higher in 1.0 × 10^6^/kg dose level (Fig. 3a and Supplementary Tables S4–5). CAR-T cells persisted in peripheral blood for up to 6 months, which could be detected in 100% (12/12), 73% (8/11), 60% (6/10), and 17% (1/6) at 1, 2, 3, and 6 months, respectively (Supplementary Table S6). Blood CAR T-cell levels showed no significant difference in patients with or without the use of steroids (Supplementary Fig. S2).

### Serum AQP4-IgG and BCMA

The serum titers of AQP4-IgG were measured using live-cell-based assay after screening. Serum AQP4-IgG for all treated participants decreased after CT103A infusion, and was negative by 12 weeks in 7 of the 10 patients (70%), and by 6 months in 5 of the 6 patients (83%) (Fig. 3b). For Patient 1, along with the decrease in CAR-T cells, the level of AQP4-IgG increased again in the serum after 8 weeks.

The serum BCMA levels significantly reduced (below lower limit of detection) within 1-month post-infusion, but gradually returned to baseline levels by 6 months (Fig. 3c).

### Efficacy

During follow-up of a median 5.5 months (range, 1–14 months), 11(92%) patients achieved drug-free remission, with no relapse with all corticosteroids and immunosuppressants discontinued,. Patient 1 had a possible attack of decreased visual acuity in the left eye at 14-month after infusion (Supplementary Fig. S3).

**Fig. 4:**
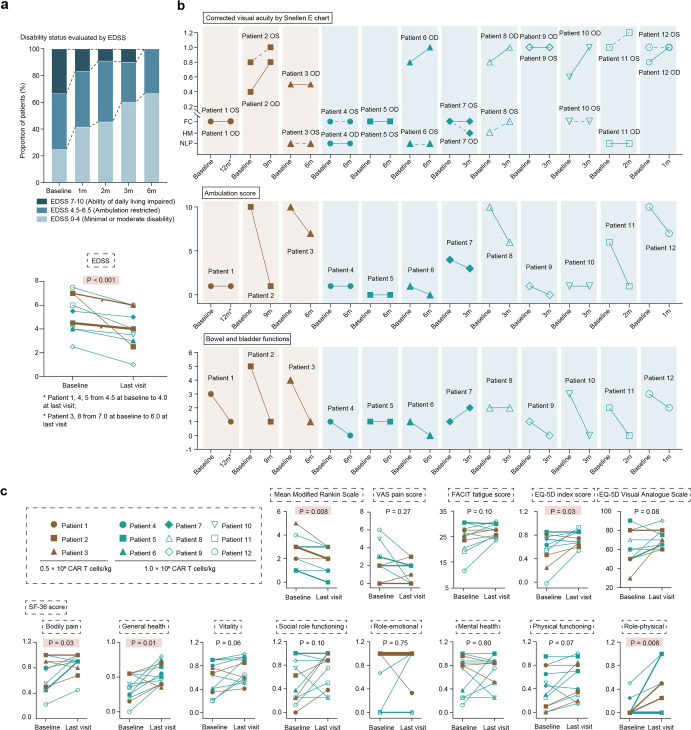
Functional improvement after CT103A infusion. **a** Distribution of EDSS score at baseline and post infusion. EDSS were shown individually at baseline and last visit. **b** Individual values of corrected visual acuity by Snellen E chart, functional system score from the Expanded Disability Status Scale in ambulation (scored from 0 [Unrestricted] to 12 [essentially restricted to bed or chair or perambulated in wheelchair]), and bowel and bladder functions (scored from 0 [no symptoms] to 6 [loss of bowel and bladder function]) at baseline and last visit. Solid and dotted lines represent changes of corrected visual acuity in oculus dexter (OD) and oculus sinister (OS), respectively. **c** Individual values of disability and quality-of-life outcomes. Secondary endpoints for disability and quality-of-life outcomes were evaluated, including the modified Rankin scale (scored from 0 [no symptoms] to 6 [death]); the Expanded Disability Status Scale (EDSS, range from 0 [normal neurologic examination] to 10 [death]), visual analog scale (VAS) score for pain (on a scale from 0 to 100, with higher scores indicating more pain); the Functional Assessment of Chronic Illness Therapy–Fatigue (FACIT-F) score (on a scale from 0 to 52, with higher scores indicating less fatigue); the EuroQol-5 Dimensions (EQ-5D) instrument (scored on a scale from −0.109 to 1, with higher scores indicating a better health state); EQ-5D-VAS (on a scale from 0 to 100, with higher scores indicating better condition); the 36-item Short Form Health Survey (SF-36; eight sections with scores transformed to 0 to 1, with lower scores indicating greater disability). Data were shown individually at baseline and last visit. Data with same values were displayed overlapped. Comparison was performed by Wilcoxon-matched-pair-signed rank test

All patients showed decrease in the EDSS score from baseline at last follow-up (Fig. 4a). 6(50%) patients reported better corrected visual acuity in at least one eye. 8(67%) patients showed improvement in ambulation, with 4 patients improved from being restricted to a wheelchair or bed to walking with or without assistance. 9(75%) patients showed improvement in bowel and bladder function, with 5 patients improving from need of manual measures to evacuate bowels or catheterization to spontaneous defecation and urination (Fig. 4b). Improvements in other endpoints for disability and quality-of-life outcomes, including the modified Rankin Scale, EuroQol-5 Dimensions, and several domains in the 36-item Short Form Health Survey were also observed (Fig. 4c and Table S7).

### Other key exploratory outcomes

**Fig. 5:**
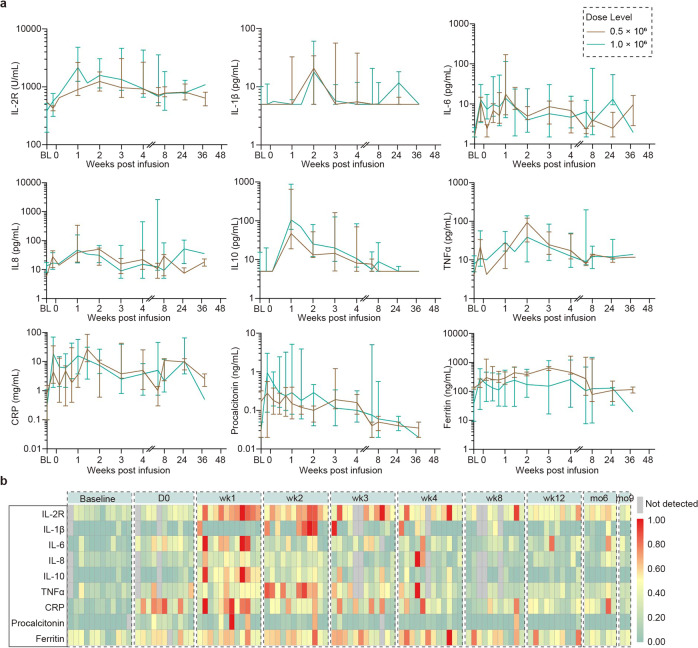
Post‑infusion levels of cytokines. **a** Kinetic changes of post-infusion values of various cytokines in median according to dose group. Bars indicate 95% confidence intervals. **b** Post-infusion fold-changes of cytokine release. IL denotes interleukin. TNF denotes tumor necrosis factor. CRP denotes C-reactive protein

A significant decrease in total immunoglobin in the serum after CT103A CAR T-Cell infusion was observed in all patients (Supplementary Fig. S4). Cytokine release peaked within 2 weeks after infusion (Fig. 5). Higher peak CAR T-cell expansion and exposure was associated with higher peak cytokine levels (Supplementary Figs. S5 and 6). A total of 3 patients (25%) had at least one sample with detectable anti-drug antibody post infusion (Supplementary Table S8).

### Co-existing autoimmune diseases

Both serologic remission and clinical improvement were observed in 2 patients with Sjögren syndrome (SS) at 12 weeks post infusion, as shown in decreased anti-SSA autoantibodies, decreased EULAR SS patient reported index and EULAR SS disease activity index, increased unstimulated salivary flow rate, and prolonged tear breakup time and Schirmer’s test. (Supplementary Table S9).

In addition, the patient with rheumatoid arthritis (RA) showed improvement in RA disease activity at 12 weeks post infusion. Disease Activity Score in 28 joints and erythrocyte sedimentation rate (DAS28ESR) decreased from 4.698 (indicating an active status) to 1.987 (indicating a remission stage), and tender joint counts decreased from 24 to 0.

## Discussion

In this ongoing phase 1, first-in-human clinical study of CT103A, CAR T-cell therapy targeting BCMA, in relapsed or refractory AQP4-IgG seropositive NMOSD, patients displayed a manageable safety profile and had a promising clinical response without any additional immunosuppressive therapy.

In our recent study, patients with MM were administrated with CT103A at different doses of 1 × 10^6^–6 × 10^6^ cells/kg. Most of them had a good response at all dose levels, complicated by severe CRS that was more common at higher doses (>3 × 10^6^ CAR T cells/kg).^10^ As a result of these toxic effects and observation of in vivo expansion of CT103A cells, the protocol for this study chose lower CT103A doses (≤1 × 10^6^ CAR T cells/kg).

AEs, mainly presented as cytopenia and infection in our study, occurred in all patients. Cytopenia are common AEs during the first few days after infusion, which might be due to lymphodepletion therapy. Both the severity and duration of cytopenia in our cohort were much lower than those in MM patients,^3,11,16,17^ possibly because myelosuppression was more serious and common in MM than NMOSD. Infection AEs were also reported, while the severity did not exceed that expected for MM patients treated with BCMA-directed CAR T cells^9^ or for NMOSD patients treated with other candidate preventive agents.^18^ Serious infections of grade 3 or higher requiring hospitalization exceeding 4 weeks occurred in 2 patients (17%). Both the patients were aged over 60 years, and had a long history of autoimmune disorders and use of immunosuppressive therapy for more than 20 years (Patient 7 with a 20-year history of NMOSD; Patient 10 with a 28-year history of RA), suggesting that elderly patients treated with long-term immunosuppressants are at a high risk of infection and need more attention. The overall frequency of treatment-related SAEs was tolerable (25%), with no progressive multifocal leukoencephalopathy or fatal events by the date cutoff.

It has been reported that 28% of the CT103A treated MM patients had CRS of grade 3 or higher,^10^ and 6–25% reported in other BCMA-directed CAR T-cell products.^11,19,20^ The severity of CRS in our cohort was much lower than that in MM, possibly owing to the lower antigen burden in NMOSD. Overall, the results observed with CT103A in NMOSD indicate a favorable safety profile. Additional data from participants with longer exposure periods are required for more accurate safety comparisons.

CAR T-cell expansion was observed in all 12 dosed patients within 14 days at both doses, and was slightly greater in those patients with a higher dose, a phenomenon similar to other studies of CAR T-cell therapy in hematologic cancer.^10,11^ Persistence was shorter than that reported in MM patients.^10^ There are two possible explanations for the discrepancy. First, abnormal plasma cells, as the antigen burden in NMOSD patients, are much less than those in MM, as the baseline levels of sBCMA in our NMOSD cohort (median 19.9 ng/mL, min–max 12.4–30.3) were much lower than those reported in MM patients (median >100 ng/mL).^10,11,19^ Second, long-term use of immunosuppressants might dampen the cellular activity of T cells, so as their CAR T-cell products. Similar limited persistence was also reported in CAR-CD19 therapy in another autoimmune disease, SLE, with a rapid decline in circulating CAR T-cell counts within 30 days post infusion.^13^ Nevertheless, studies have demonstrated that the durability of remission in hematologic malignancies is not definitely associated with the long-term persistence of CAR T cells.^21^ Drug-free remission was also reported in the 5 patients with SLE who received CAR T-cell therapy, despite CAR-CD19 cells diminished about 30 days after infusion and B-cell reconstitution occurred about 3 months post-treatment.^13^ In this NMOSD cohort, sustained inhibition of humoral immunity, as well as low levels of AQP4-IgG in the serum, were observed. Most patients showed no infection without regular replacement therapy, suggesting that sustained humoral immunity suppression was well tolerated in NMOSD patients.

During the follow-up of a median 5.5 months, we observed reduced attack frequency, and stabilized or improved neurological disability measures in most patients, indicating a preliminary clinical efficacy of CAR-BCMA therapy in NMOSD. The possibility that the lymphodepletion conditioning regimen before CT103A infusion was responsible for an apparent decrease in relapses should also be considered. Lymphodepletion therapy with combination of cyclophosphamide plus fludarabine was wildly used for CAR T-cell expansion and activity in hematological malignancies, which was also adopted in a recent study of CAR T-cell therapy for SLE.^13^ However, influences from cyclophosphamide are likely small and short-lived, given several adverse biological features of the enrolled participants’ NMOSD, including highly aggressive status, severe disability, and resistance to multiple prior immunosuppressive therapies. Whether fludarabine has therapeutic effects on NMOSD warrants further investigation. In addition, pain, fatigue and depressive state are frequent in NMOSD and can constitute a significant burden.^22,23^ Patients in this study generally reported improvements in these areas. Although before-after differences were observed in several measures of quality of life, the small sample size and the brief observation limits the interpretation of the clinical outcomes related to efficacy. Nevertheless, our data show that CAR-BCMA T-cell therapy might induce clinical remission of NMOSD associated with the rapid removal of AQP4 autoantibodies, and longer-term observation will be warranted.

Preclinical data indicate that AQP4-IgG triggers amplification of inflammatory response, antibody-dependent and complement-dependent cellular cytotoxicity that can lead to potential injury of astrocytes, surrounding cells, myelin and axons.^1^ Based on the pathophysiology of NMOSD, satralizumab/tocilizumab (anti-IL-6 receptor), eculizumab (anti-complement protein C5), rituximab (anti-CD20^+^ B cells) and inebilizumab (anti-CD19^+^ B cells) were recently proved to be effective in preventing attacks for NMOSD.^3,5,6,24,25^ Despite the increasing progress in the treatment of NMOSD, there are still a subset of patients do not respond to these therapies and suffer from recurrence, severe sequelae, and even death.^18^ Direct comparison of our CAR T-cell therapy and these “mabs” in terms of efficacy is difficult because there are great differences in trial design and inclusion criteria. In addition, rapid recurrence of attacks were observed after “mab” therapy cessation or during long treatment intervals,^18^ making it essential to assess durability of CT103A in NMOSD patients. While post-infusion anti-drug antibody was detected in 25% patients, which might restrict a second infusion, long-term follow-up data will determine that whether CAR T cells monotherapy would be a promising therapeutic option for durable disease control in AQP4-IgG seropositive NMOSD, differentiating it from other therapies that require repeated and/or continuous administration, with or without concomitant immunosuppressive therapy.

Moreover, patients with AQP4-IgG-positive NMOSD are often complicated with other autoimmune diseases, such as SS, and RA.^1^ Given the role of plasma cells in various severe autoimmune diseases, our observation of clinical remission in co-existing autoimmune disorders, and a recent report of anti-CD19 CAR T-cell therapy in SLE,^13^ advances in CAR T technology have the potential to broaden CAR-engineered T-cell therapies and foster new applications beyond oncology in autoimmunity.^26^ Another expansion phase is currently underway to determine the safety and efficacy of CAR T-BCMA infusion in patients with other neuro-inflammatory diseases.

This study has several limitations. First, newly approved therapies including eculizumab, satralizumab and inebilizumab were not available or approved for treating NMOSD in China at the time of the initiation of the trial. None of the included patients were ever treated with this medication. A parallel treatment arm with these approved therapies will be set in the future exploration. Second, the follow-up duration is brief, significantly limiting the interpretation of the clinical outcomes. Longer evaluation is continued. Further, the non-randomized design and the small patient number in this trial restrict the conclusions drawn. Lastly, CAR T cells specific for the B cells producing anti-AQP4 antibody or directly targeting the AQP4-IgG would be more accurate for the disease control, which warrant further investigation.

This first-in-human study has provided an initial proof-of-concept that CAR-BCMA T cells, CT103A, could serve as a safe and potential monotherapy for patients with relapsed or refractory AQP4-IgG seropositive NMOSD, and warrant further evaluation in wider applications, especially for antibody-mediated autoimmune diseases.

## Methods

### Study design and oversight

This is an investigator-initiated, open-label, single-arm, phase 1 study, which is conducted at Tongji Hospital, Tongji Medical College, Huazhong University of Science and Technology, China. The study was registered at ClinicalTrials.gov, NCT04561557, and the protocol was approved by the Institutional Review Board of Tongji Hospital. All patients provided written informed consents before screening.

A detailed treatment plan is provided in the study protocol (Supplementary data). Briefly, leukapheresis was performed to acquire sufficient autologous peripheral-blood mononuclear cells (PBMCs). The CAR-BCMA cells, CT103A, was manufactured by IASO Biotherapeutics from PBMCs, transduced with a lentiviral vector containing a fully human anti-BCMA single-chain fragment variable, a CD8a hinge and transmembrane, 4-1BB co-stimulating domain, and the CD3- ζ T-cell activation domain, as described in our recent studies.^10^ No sorting was performed post transduction. Transfection efficiency was detected by flowcytometry as percentage of infused CD3^+^ cells expressing CAR-BCMA. Characterization of CAR T-cell infusion products were shown in Table S1.

To allow CAR T-cell expansion, all patients were given lymphodepletion therapy, consisted of cyclophosphamide 500 mg/m^2^ plus fludarabine 30 mg/m^2^, on days –4, –3, and –2. CT103A was infused on day 0. The study consisted of two parts (Fig. 1): doses of 0.5 × 10^6^ and 1.0 × 10^6^ total CAR T cells per kilogram body-weight (±20%) were tested in the dose-escalation phase and 1.0 × 10^6^ CAR T cells/kg in the expansion phase. The cutoff date for this month 5.5 interim analysis was on March 20, 2022. The primary outcome was safety. The follow-up to assess durability will continue for 2 years.

The sample size was based on a traditional 3 + 3 dose-escalation design and clinical considerations based on our previous study on CT103A in MM.^10^

### Patients

The main eligibility criteria for this study were an age of 18–75 years; a diagnosis of NMOSD, according to the 2015 International Panel for Neuromyelitis Optica Diagnosis criteria;^27^ AQP4-IgG–seropositive status confirmed with a microscopic live-cell-based assay (CBA) as previously reported;^28^ Full-dose medication of at least one immunosuppressant for more than one year, with relapses uncontrolled; More than two relapses within the previous 12 months or three relapses within the previous 24 months with one of which occurred within the previous 12 months.

Patients with rituximab treatment within 5 months, or plasma exchange and immunoglobulin treatment within 4 weeks prior to screening were not enrolled. Immunosuppressants and oral corticosteroids were stopped before lymphodepletion. Detailed description is provided in the study protocol (Supplementary data).

### Study procedures and endpoints

The primary endpoint of this study was safety. CRS and ICANS was defined and graded according to published criteria.^29,30^ Adverse events (AEs) were assessed by investigators according to the National Cancer Institute Common Terminology Criteria for Adverse Events, version 5.0. Relapses were not categorized as AEs.

Secondary endpoints were defined as CT103A levels in blood and circulating serum AQP4 antibodies levels detected by CBA. Key exploratory outcomes included efficacy, disability and life quality, measurement of select cytokines, serum BCMA, and anti-drug antibody testing.

### Flow cytometric analysis

CAR T-cell percentage in CD3^+^ T lymphocytes were detected by flow cytometric analysis (MACS Quant Analyzer 10). FITC-Labeled Human BCMA Fc Tag protein (BCA HF254, Acro Company) were used for detecting CAR^+^ cells, APC Mouse Anti-human CD45 antibody (8930264, Agilent) for lymphocytes, PerCP Mouse Anti-human CD3 antibody (8931015, Agilent) for CD3-positive T cells.

### Droplet digital PCR (ddPCR)

The CAR transgene copies were detected by droplet digital polymerase chain reaction (ddPCR) with probe and primers targeting the scFV sequence as described in our previous study.^10,31^

### Statistical analysis

Data are provided on individual patients in tables and figures. Descriptive summaries are reported as medians (ranges) and counts (percentages). Paired comparisons between two timepoints were performed with Wilcoxon-signed rank test. Analyses were done in SAS, version 9.4.

## Supplementary information

Supplementary material

supplemantary figure 1

supplemantary figure 2

supplemantary figure 3

supplemantary figure 4

supplemantary figure 5

supplemantary figure 6

supplemantary figure 7

supplemantary protocol

## Data Availability

Dr. Wei Wang had full access to all the data in the study and takes responsibility for the integrity of the data and the accuracy of the data analysis. Individual participant data that underlie the results reported in this article, after de-identification, will be shared within 5 years upon publication. Data will be available after approval of a proposal with a signed data access agreement to achieve aims in the approved prospectus. Proposals should be directed to wwang@tjh.tjmu.edu.cn.
