# Supplementary material for: Anti-BCMA CAR T-cell therapy CT103A in relapsed or refractory AQP4-IgG seropositive neuromyelitis optica spectrum disorders: phase 1 trial interim results
Source: Signal Transduct Target Ther. 2023 Jan 4;8:5. doi: 10.1038/s41392-022-01278-3 (PMC9810610; doi:10.1038/s41392-022-01278-3)
Supplement: Supplementary file 1 — Supplementary material [file 41392_2022_1278_MOESM1_ESM.docx]

Supplementary Materials for

Anti-BCMA CAR T-Cell Therapy CT103A in Relapsed or Refractory AQP4-IgG Seropositive Neuromyelitis Optica Spectrum Disorders: phase 1 trial interim results

Subtitle: CAR T-BCMA Therapy in NMOSD

Chuan Qin^1,2^ *; Dai-Shi Tian^1,2^ *; Luo-Qi Zhou^1,2^; Ke Shang^1,2^; Liang Huang^3^; Ming-Hao Dong^1,2^; Yun-Fan You^1,2^; Jun Xiao^1,2^; Ying Xiong^4^; Wen Wang^5^; Hao Pang^5^; Jing-Jing Guo^5^; Song-Bai Cai^5^; Di Wang^3^; Chun-Rui Li^3^; Min Zhang^1,2^; Bi-Tao Bu^1,2^; Wei Wang^1,2^

Correspondence to: Wei Wang MD, PhD E-mail: wwang@tjh.tjmu.edu.cn

**This PDF file includes:**

Materials and Methods

Supplementary Text

Figures. S1 to S7

Tables S1 to S9

Materials and Methods

**Study design and oversight**

The sponsor, IASO Biotherapeutics (Nanjing, China), designed the trial in consultation with five academic authors, and provided the trial agents. All authors vouch for the fidelity of the trial to the protocol as well as for the accuracy and completeness of the reporting of results and adverse events as stipulated in the protocol. Confidentiality agreements were in place between the authors and sponsor.

Lymphodepletion therapy are as follows: Cyclophosphamide 500mg/m^2^ IV over 30 minutes, once daily for 3 consecutive days; Fludarabine 30mg/m^2^ IV over 30 minutes (immediately after cyclophosphamide infusion is completed), once a day for 3 consecutive days. Patients received lymphodepletion therapy on days –4, –3, and –2, followed by an infusion of CT103A on day 0.

**Cytokine release syndrome (CRS)**

CRS is due to the release of inflammatory cytokines leading to syndrome of symptoms such as fever, nausea, headache, tachycardia, hypotension, rash, and shortness of breath. ^1^ The standard for CRS classification (CRS grading standard recommended by ASTCT in 2019) in this study uses fever, blood pressure, and blood oxygen saturation for CRS grading (see protocol in Supplement 1 for the grading standard). The indications and precautions of tocilizumab and glucocorticoids in the treatment of CRS are decided by the treating physicians according to the protocol.

**Dose Limiting Toxicity (DLT)**

DLT is defined as CTCAE of Grade 3-5 (CTCAE version 5.0) newly occurring within 28 days after CT103A infusion, which are related to CT103A treatment (definitely related, likely related, possibly related). EXCEPT FOR the following events:

1. Grade ≥ 3 CRS and neurotoxicity that can be relieved to Grade ≤ 2 within 3 days after supportive therapy;
2. Hematological toxicity including Grade 3 neutrophil count decrease of any duration or Grade 4 neutrophil count decrease lasting < 28 days; Grade 3 anemia of any duration or Grade 4 anemia lasting < 28 days; Grade 3 platelet count decrease of any duration or Grade 4 platelet count decrease lasting < 28 days; Decreased peripheral blood counts other than the above.
3. Grade ≥ 3 non-hematologic toxicities that resolve to Grade ≤ 2 within 7 days.

**NMOSD Relapse definition**

Protocol-defined relapses were new or worsening subjective symptoms and objective findings on neurological examination with one of the following as previously described^2^:

1. an increase of more than 1.0 on the Expanded Disability Status Scale (EDSS);
2. an increase of at least 2.0 on one appropriate symptom-specific functional-system score for the pyramidal system, cerebellar system, brain stem, sensory system, bowel or bladder, or a single eye;
3. an increase of at least 1.0 on more than one symptom-specific functional-system score;
4. an increase of at least 1.0 on a symptom-specific functional-system score in a single eye.

In addition, symptoms were required to be attributable to NMOSD attacks, and persist for more than 24 hours, and without interfering by confounding factors such as infection, trauma, change in mood, or adverse events.

**Serum AQP4-IgG measurement**

Only AQP4-IgG–seropositive NMOSD patients, confirmed by live cell-based assay (CBA, including seronegative individuals who had previously been seropositive), were included in this study. The serum levels of AQP4-IgG were measured using CBA at visits defined in protocol after screening.

**Anti-drug antibody (ADA) detection assay**

The anti-drug antibody of CT103A was evaluated by ECL bridging assay on Meso Scale Discovery Electrochemiluminescence (MSD-ECL) platform (MSD, Gaithersburg, MD, USA) as described in our previous study. ^3^ A multi-staged ADA testing approach was used to detect ADA, including screening assay, confirmatory assay, and titration assay. Positive samples in the screening assay would be further detected by confirmatory assay to test the ADA specificity, and then their titers would be determined by titration assay.

**Secondary and key exploratory outcomes**

Secondary end points were quantification of CT103A in blood and circulating serum AQP4 antibodies. Key exploratory outcomes included efficacy (measured by number of attacks), disability (measured by EDSS, range from 0 [normal neurologic examination] to 10 [death]), measurement of select cytokines, serum BCAM, and anti-drug antibody testing. Additional secondary efficacy end points were the change from baseline to week 12/month 6 and last visit on the following assessments: visual analogue scale (VAS) score for pain (on a scale from 0 to 100, with higher scores indicating more pain); the Functional Assessment of Chronic Illness Therapy–Fatigue (FACIT-F) score (on a scale from 0 to 52, with higher scores indicating less fatigue); the 36-item Short Form Health Survey (SF-36; eight sections with scores transformed to 0 to 100, with lower scores indicating greater disability); the EuroQol-5 Dimensions (EQ-5D) instrument (scored on a scale from −0.109 to 1, with higher scores indicating a better health state); EQ-5D-VAS (on a scale from 0 to 100, with higher scores indicating better condition); the modified Rankin scale (scored from 0 [no symptoms] to 6 [death]).

The ambulation score, an important functional system score of EDSS measurement, represents both a description of walking range and the type of assistance required for ambulation, range from 0 (Unrestricted) to 12 (essentially restricted to bed or chair or perambulated in wheelchair).

**Clinical outcomes for Sjögren’s Syndrome and rheumatoid arthritis**

Clinical outcomes for Sjögren’s Syndrome included EULAR Sjogren’s syndrome disease activity index (ESSDAI)^4^, patient-reported outcomes included the EULAR Sjögren’s Syndrome Patient Reported Index (ESSPRI)^5^, with higher scores indicating greater disease activity. Functional clinical outcomes of disease activity included stimulated and unstimulated salivary flow rate, tear breakup time, and Schirmer’s test results^6^. Clinical outcomes and disease activity of rheumatoid arthritis were assessed with the Disease Activity Score for 28 joints (DAS28) with the use of the erythrocyte sedimentation rate (DAS28-ESR), with higher scores indicating greater disease activity.^7^

Supplementary Text

Subhead

**Key secondary and exploratory outcomes**

***Serum BCMA (sBCMA)***

The sBCMA levels significantly reduced within 1-month post-infusion (**Fig. 3c**), suggesting effective depletion of plasma cells. The extent of reduction did not differ between the two doses. The sBCMA levels in all 12 patients were below lower limit of detection (3.2 ng/mL) by 4 weeks, but approximately returned to baseline levels by 6 months. Interestingly, AQP4-IgG remained undetected in 5 of the 6 patients (83%), even when their sBCMA levels returned to baseline levels.

***Lymphocyte subsets***

Percentage of CD3^-^CD19^+^ B cells, CD3^+^CD19^-^ T cells and CD3^-^CD16^+^CD56^+^ NK cells in lymphocytes were detected by flow cytometry in all patients before and after CAR T cells infusion. CD19^+^ B lymphocytes were undetectable in all patients within 4 weeks after infusion, which was expected to result from both lymphodepleting chemotherapy and CAR T cells expansion, and returned within 12 weeks (**Supplementary Fig. S1**).

***Immunoglobin in serum***

A significant decrease in total immunoglobin in the serum after CT103A CAR T-Cell infusion was observed in all 12 patients (**Supplementary Fig. S4**). Single dose of intravenous immunoglobulin was routinely administered in patients with IgG ≤ 5 g/L in the case of or for the treatment of viral infection in the first 3 months after infusion. Immunoglobulin was administered for a second time in 3 of the 10 patients (30%) during long-term follow-up (from 12 weeks post-infusion to the cutoff date; 1 for fever of unknown reason, 1 for CMV infection, and 1 for severe erythema multiforme, all resolved in 1 week). There was no evidence of long-term or regular intravenous immunoglobulin replacement in any of the patients.

***Serum Cytokine***

Cytokine release, including IL-2R, IL-1β, IL-6, IL-8, IL-10, TNF-α, C-reactive protein, procalcitonin, and ferritin, peaked between 1 to 2 weeks after infusion (**Fig. 5**). Several patients showed another increase in serum cytokines levels after the peak, correlated with their infection. The second increase all returned to baseline and the infections resolved within 1 month.

Higher peak CAR T-cell expansion was associated with higher peak levels of serum IL-2R, IL-10, IL-6 and C-reactive protein; Larger exposure was associated with higher peak levels of serum IL-2R, CRP, and ferritin. No correlation was observed between baseline levels of sBCMA with CAR T-cell expansion and exposure (**Supplementary Fig. S7**).

***Anti-drug antibody (ADA)***

A total of 3 patients (25%) had at least one sample with detectable ADA post infusion (Supplementary **Table S8**). Among them, Patient 1 were found to have very low titer of pre-existing ADA before CT103A infusion, transiently turned to be negative at 1 month post infusion, and then returned to be positive at 3 months with the titer keeping increasing to as high as 3884.1 at month 10. For Patient 2 and 5, ADA emerged at 6 months post infusion and the titers were 5.7 and 6.3, respectively, and 10.5 for Patient 2 at 9 month.

**Case presentation**

**Detailed information of Patient 1**

Patient 1, a 31-year-old man, experienced eight relapses under regular immunosuppressant therapy, including azathioprine, mycophenolate mofetil, tacrolimus, and rituximab (**Supplementary Table S1**). He had severe fungal pneumonia requiring intensive care treatment 1 week after the fifth infusion of rituximab. The EDSS score was 4.5 at baseline. After CT013A infusion, the patient had grade 1 CRS, and cytopenia of grade 4 which resolved within 4 weeks. No other AEs of grade 3 or higher occurred.

However, along the decrease in CAR-T cells, the level of serum AQP4-IgG increased in this patient after 9 weeks. A second infusion of 1.0×10^6^ total CT103A cells was administered at 9 months after the first dose with patient’s agreement and according to the protocol. But no expansion was observed this time. In a post hoc analysis of ADA, Patient 1 were the only one found to have pre-existing ADA before CT103A infusion as described above. Notably, a sudden rise of ADA occurred almost simultaneously with the second infusion of CT103A in Patient 1, which might explain the failed expansion of the second infusion (Supplementary **Fig. S3**). Several lines of evidence have indicated that potential immunogenicity against CAR T cells *in vivo* may cause the elimination of certain CAR T cells,^8,9^ which is marked by the very limited success in re-dosing CAR T-cell products in various trials. ^10,11^ Whether the presence of pre-treatment ADA affects the activity of CAR T cells is still controversial.^12,13^ Meanwhile, our previous study suggested that such emergence of ADA may be associated with the loss of CAR T cells *in vivo*.^3^ In addition, post-infusion ADA was detected in 3 patients (25%), the percentage of which was higher than that in MM patients. ^3^ Autoimmune diseases with pathologically enhanced humoral immunity might explain this phenomenon. Patient 1 was the only one patient received a second dose in our cohort.

Patient 1 had a possible attack at 14-month after infusion. The patient complained of decreased visual acuity in the left eye, without objective evidence of relapse, including no corresponding lesion changes on optic nerve magnetic resonance and visual evoked potential changes. High-dose steroids were administered and oral prednisone (20 mg) was maintained thereafter. Patient 1 was the only one patient had a relapse during the follow-up.

**Detailed information referring to co-existing autoimmune diseases**

Of the 3 patients with Sjögren syndrome (SS), improvements in both clinical outcomes and laboratory measures were observed in 2 patients (Patient 12 did not reach her 12-week follow-up by the cutoff date). The levels of anti-SSA autoantibodies (a determined pathogenetic antibody in SS) decreased rapidly from 28.04 U/mL to below the minimum detection level in Patient 9 and to from 291.69 U/mL to 2.29 U/mL (indicating a negative status) in Patient 10 within 12 weeks. These signs of serologic remission were parallel to those of clinical remission. At enrolment and 12-week follow-up, the EULAR Sjögren's Syndrome patient reported index (ESSPRI) and EULAR Sjögren's syndrome disease activity index (ESSDAI) were calculated as previously defined.^14^ At week 12, a decrease was observed in the level of ESSPRI, from 11 to 3 in Patient 9 and from 17 to 8 in Patient 10. Similar changes were observed in the ESSDAI, with the score decreasing from 5 to 4 in Patient 9, and 11 to 8 in Patient 10. In correlation with these changes, the unstimulated salivary flow rate increased, together with tear breakup time and Schirmer’s test prolonged. (**Supplementary Table S9**).

In addition, the patient with rheumatoid arthritis (RA, Patient 10) showed improvement in RA disease activity at 12 weeks post infusion. Disease Activity Score in 28 joints and erythrocyte sedimentation rate decreased from 4.698 (indicating an active status) to 1.987 (indicating a remission stage), and tender joint counts decreased from 24 to 0 in Patient 10.

**References:**

1 Lee, D. W. *et al.* Current concepts in the diagnosis and management of cytokine release syndrome. *Blood*. **124**, 188-195, (2014).

2 Yamamura, T. *et al.* Trial of Satralizumab in Neuromyelitis Optica Spectrum Disorder. *N Engl J Med*. **381**, 2114-2124, (2019).

3 Wang, D. *et al.* A phase 1 study of a novel fully human BCMA-targeting CAR (CT103A) in patients with relapsed/refractory multiple myeloma. *Blood*. **137**, 2890-2901, (2021).

4 Seror, R. *et al.* EULAR Sjogren's syndrome disease activity index: development of a consensus systemic disease activity index for primary Sjogren's syndrome. *Ann Rheum Dis*. **69**, 1103-1109, (2010).

5 Seror, R. *et al.* EULAR Sjogren's Syndrome Patient Reported Index (ESSPRI): development of a consensus patient index for primary Sjogren's syndrome. *Ann Rheum Dis*. **70**, 968-972, (2011).

6 Dorner, T. *et al.* Treatment of primary Sjogren's syndrome with ianalumab (VAY736) targeting B cells by BAFF receptor blockade coupled with enhanced, antibody-dependent cellular cytotoxicity. *Ann Rheum Dis*. **78**, 641-647, (2019).

7 Taylor, P. C. *et al.* Baricitinib versus Placebo or Adalimumab in Rheumatoid Arthritis. *N Engl J Med*. **376**, 652-662, (2017).

8 Lamers, C. H. *et al.* Immune responses to transgene and retroviral vector in patients treated with ex vivo-engineered T cells. *Blood*. **117**, 72-82, (2011).

9 Maus, M. V. *et al.* T cells expressing chimeric antigen receptors can cause anaphylaxis in humans. *Cancer Immunol Res*. **1**, 26-31, (2013).

10 Maude, S. L. *et al.* Chimeric antigen receptor T cells for sustained remissions in leukemia. *N Engl J Med*. **371**, 1507-1517, (2014).

11 Lee, D. W. *et al.* T cells expressing CD19 chimeric antigen receptors for acute lymphoblastic leukaemia in children and young adults: a phase 1 dose-escalation trial. *Lancet*. **385**, 517-528, (2015).

12 Xu, J. *et al.* Exploratory trial of a biepitopic CAR T-targeting B cell maturation antigen in relapsed/refractory multiple myeloma. *Proc Natl Acad Sci U S A*. **116**, 9543-9551, (2019).

13 Mueller, K. T. *et al.* Clinical Pharmacology of Tisagenlecleucel in B-cell Acute Lymphoblastic Leukemia. *Clin Cancer Res*. **24**, 6175-6184, (2018).

14 Park, E. H. *et al.* Baseline disease activity influences subsequent achievement of patient acceptable symptom state in Sjogren's syndrome. *Rheumatology (Oxford)*. **60**, 2714-2724, (2021).


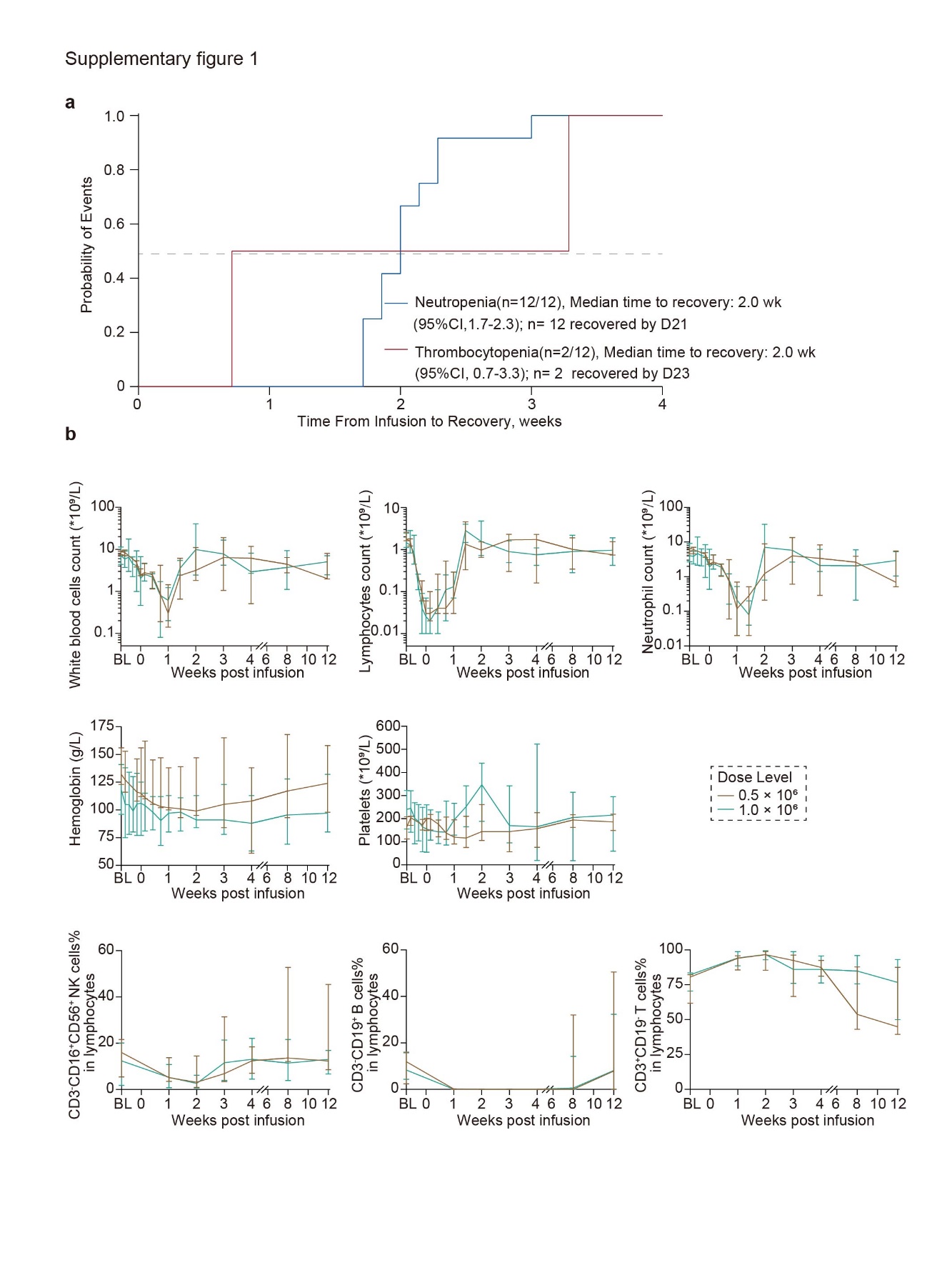


Figure. S1.

**Time to Recovery of Grade 3/4 Cytopenias and cellular kinetics post infusion.**

**(a)** Patients with grade 3/4 cytopenias (absolute neutrophil counts <1000 cells/µL or platelets <50,000/µL) on or before month 1 are included. Recovery is defined as absolute neutrophil counts ≥1000 cells/µL and platelets ≥50,000 cells/µL. Time to recovery is defined as the time from infusion to the first time when recovery criteria were met. Median and 95% CI are from Kaplan-Meier estimates.

**(b)** shows cellular kinetics as measured by median white blood cells, lymphocytes, neutrophil, hemoglobin, platelets, and percentage of CD3^-^CD19^+^ B cells, CD3^+^CD19^-^ T cells and CD3^-^CD16^+^CD56^+^ NK cells in lymphocytes, according to dose group. Bars indicate 95% confidence intervals.


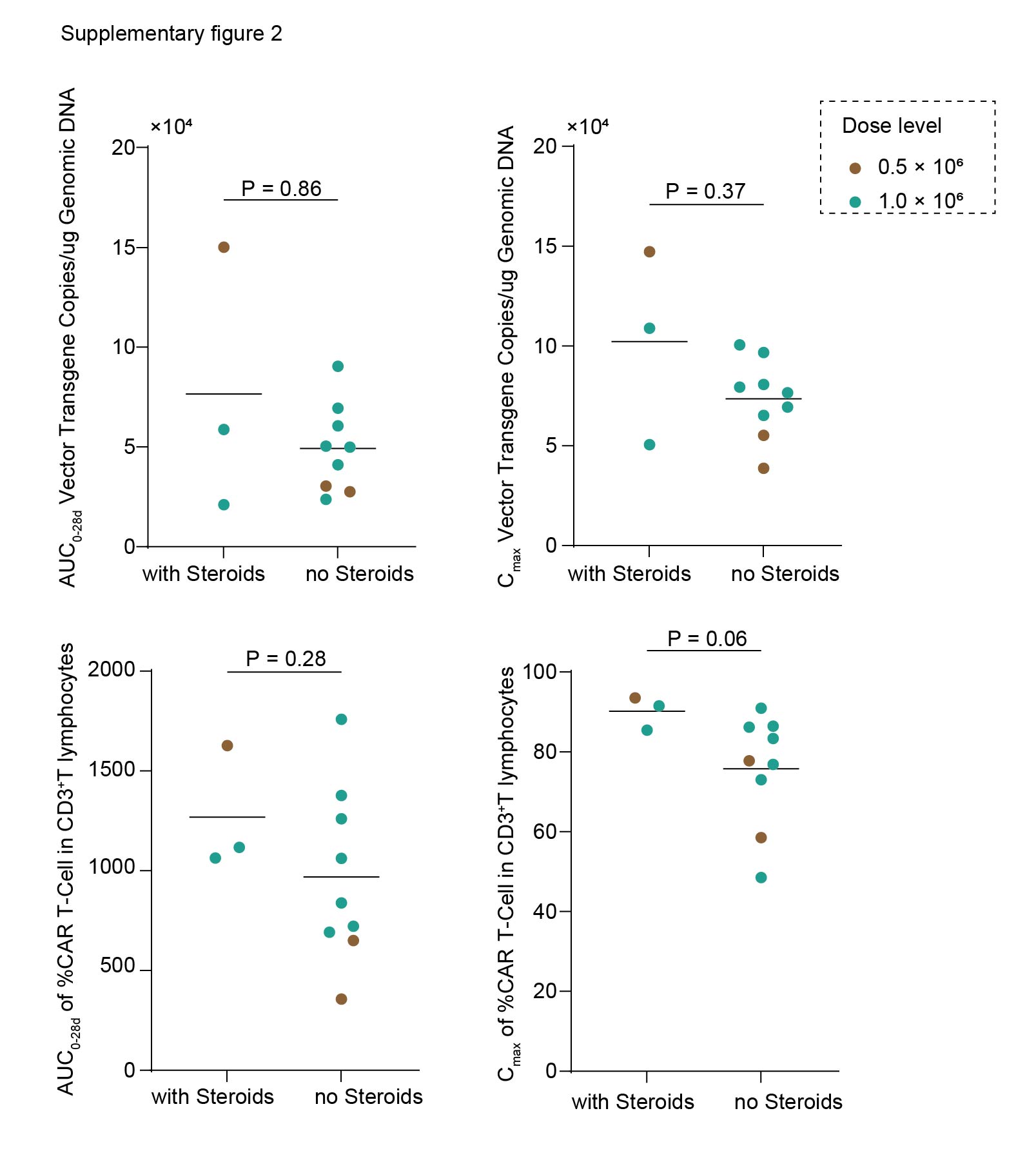
Figure. S2. Comparison of CAR transgene and CAR+ T cell percentage kinetics according to steroid treatment

AUC0-28d and peak of vector transgene copies per microgram genomic DNA and CAR+ T cells percentage in CD3+ T lymphocytes are presented in patients with steroids, and patients without steroid treatment. Horizontal lines indicate the medians. Circles indicate individual patients according to dose. The P value is based on the 2-sided Wilcoxon rank-sum test. AUC0-28d denotes area under the curve during the first 28 days after infusion. Cmax denotes maximum concentration.


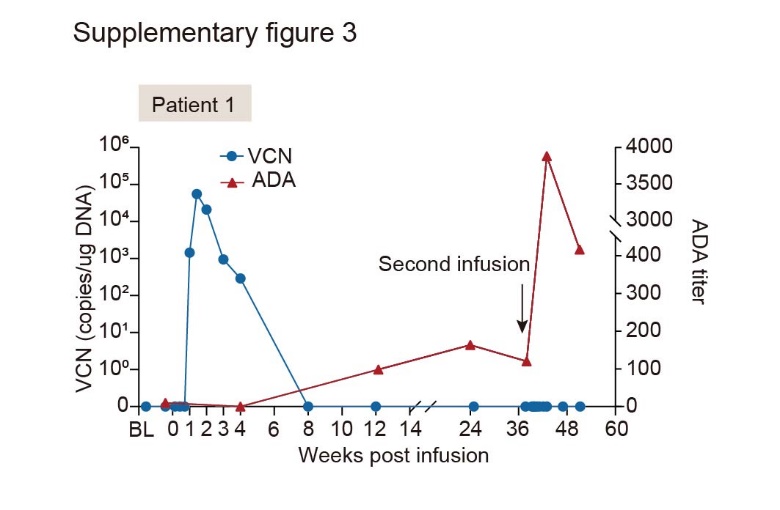


Figure. S3. Case presentation for Patient 1

Detailed data of Patient 1 post infusion. Post‑infusion values of ADA and CAR transgenes in Patient 1. Notably, a sudden rise of ADA occurred almost simultaneously with the second infusion of CT103A in Patient 1, which failed to expand for the second time.


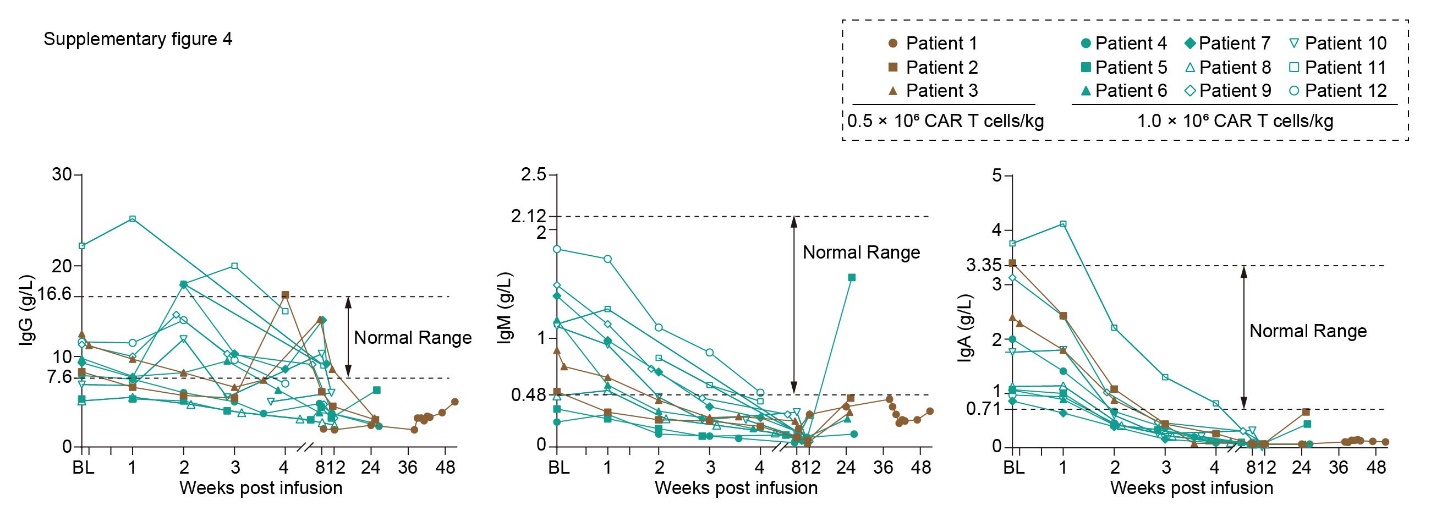


Figure. S4. Post‑Infusion Levels of immunoglobin in serum

Kinetic changes of post-infusion values of different types of immunoglobin in serum were shown individually.


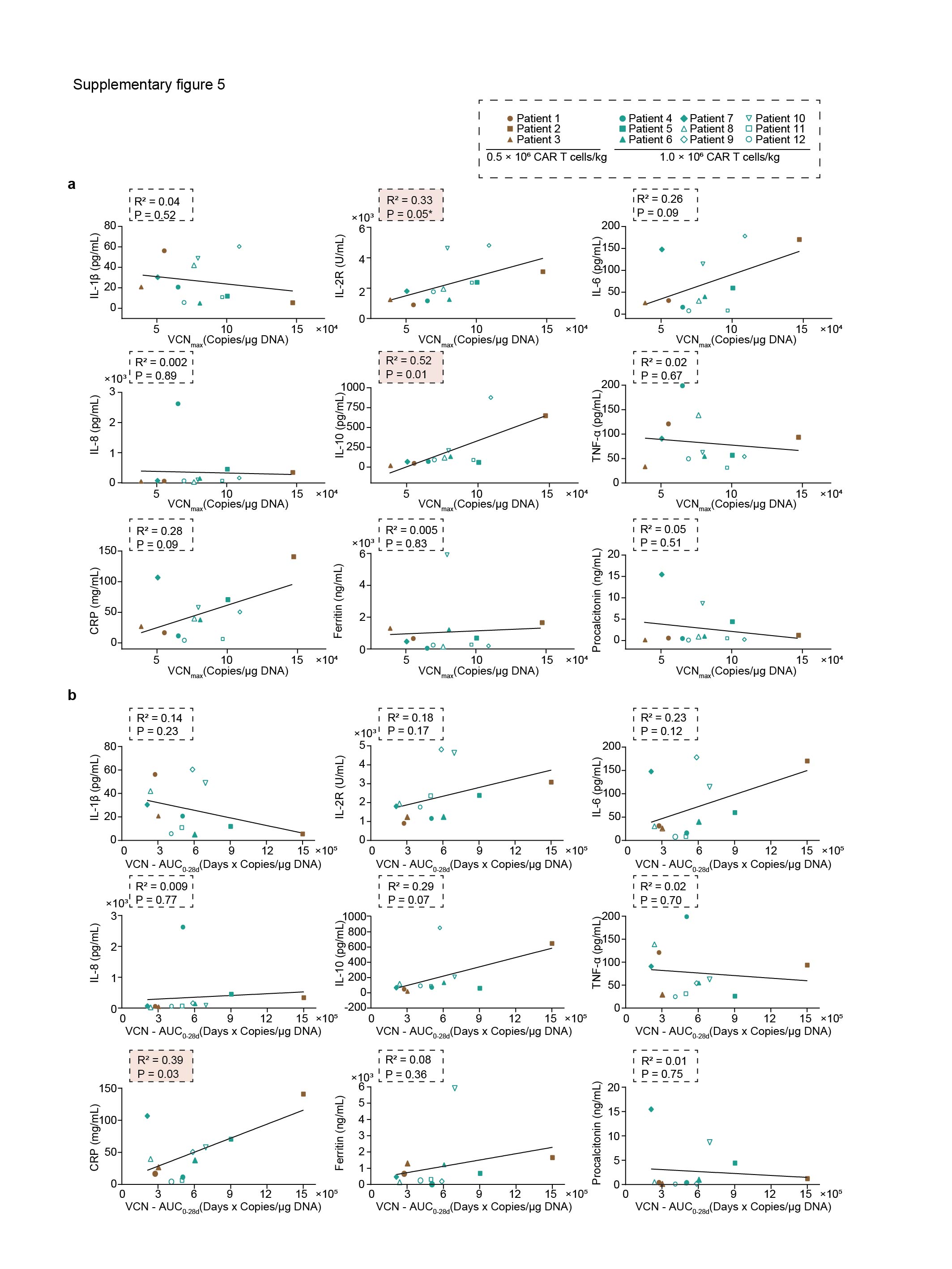


Figure. S5. Correlation analysis between cytokine release and CAR T-Cell expansion in vector transgene copies

Correlation analysis between peak levels of several cytokines and CAR T-Cell expansion (peak and AUC_0-28d_ of vector transgene copies per microgram genomic DNA) was performed by linear regression analysis.

VCN denotes viral copy number. C_max_ denotes maximum concentration. IL denotes interleukin. TNF denotes tumor necrosis factor. CRP denotes C-reactive protein. AUC_0-28_ denotes area under curve from 0 to 28 days post infusion. *The P value is 0.0496.

**
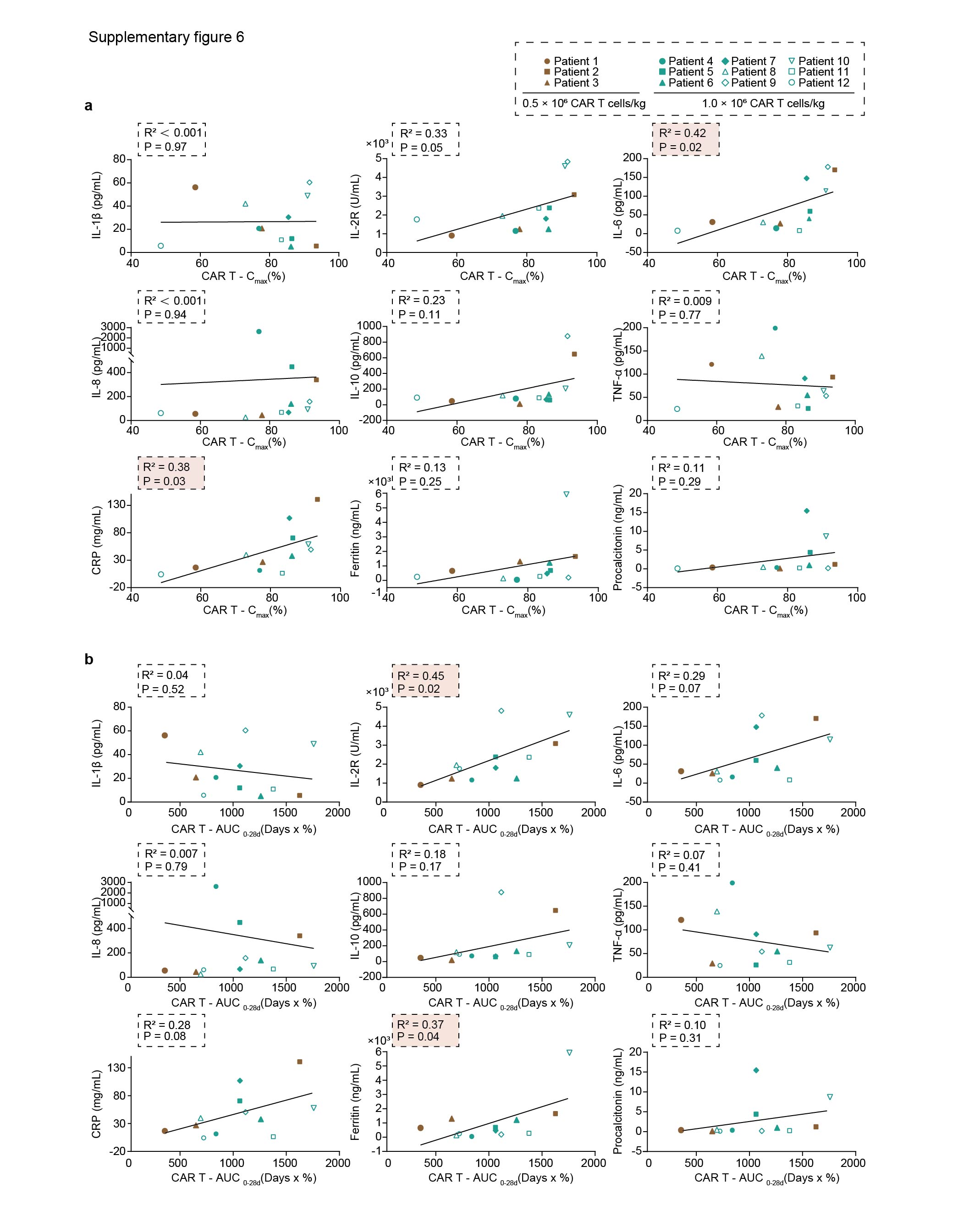
**

Figure. S6. Correlation analysis between cytokine release and CAR T-Cell expansion in CAR T-cell percentage in lymphocytes

Correlation analysis between peak levels of several cytokines and CAR T-Cell persistence (peak and AUC_0-28d_ of CAR T-Cell percentage in lymphocytes) was performed by linear regression analysis.

VCN denotes viral copy number. AUC_0-28d_ denotes area under the curve during the first 28 days after infusion. IL denotes interleukin. TNF denotes tumor necrosis factor. CRP denotes C-reactive protein. AUC_0-28_ denotes area under curve from 0 to 28 days post infusion.


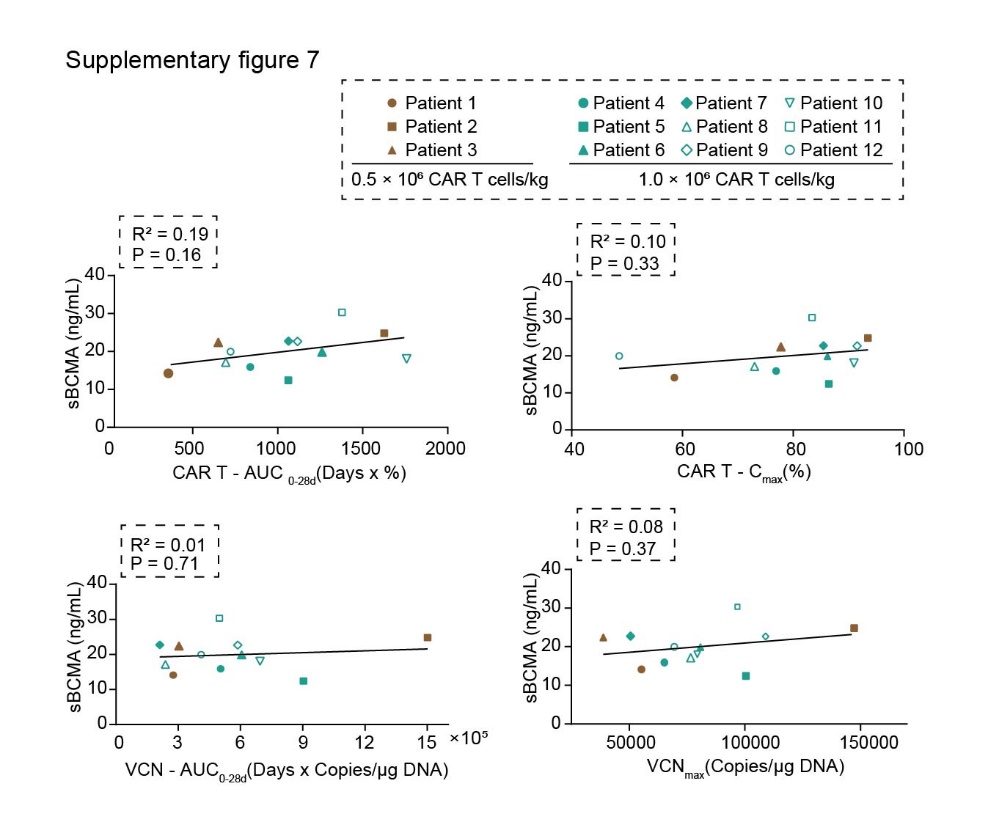


Figure. S7. Correlation analysis between the baseline levels of serum BCMA and the CAR T-Cell expansion

Correlation analysis between the baseline levels of serum BCMA with CAR T-Cell expansion (peak of vector transgene copies per microgram genomic DNA and CAR T-Cell percentage in lymphocytes, AUC_0-28d_ of vector transgene copies per microgram genomic DNA and CAR T-Cell percentage in lymphocytes) was performed by linear regression analysis.

VCN denotes viral copy number. C_max_ denotes maximum concentration. AUC_0-28d_ denotes area under the curve during the first 28 days after infusion.

Table S1.

|  | | Table S1. Additional Baseline Characteristics of the dosed Population and the infused CAR T-cells | | | | | | | | | | |
| --- | --- | --- | --- | --- | --- | --- | --- | --- | --- | --- | --- | --- |
| Patient No. | Age | | Gender | Duration of disease (months) | Long-term oral corticosteroids | Previous maintenance therapy | EDSS at baseline | AQP-4 IgG Titer before screening  (CBA) | sBCMA before screening (ng/mL) | CAR T cells/kg (10^6^) | Transduction efficiency^a^ | Days of culture |
| 1 | 31 | | Male | 52 | Y | AZA→ FK506→ MMF→ Rituximab→ FK506→ AZA | 4.5 | 1:320 | 14.09705 | 0.5 | 51% | 9 |
| 2 | 60 | | Female | 27 | Y | FK506 | 7.0 | 1:1000 | 24.79371 | 0.5 | 59% | 9 |
| 3 | 57 | | Female | 24 | Y →discontinued for glucocorticoid-induced osteonecrosis | FK506 | 7.0 | 1:3200 | 22.40334 | 0.5 | 68% | 11 |
| 4 | 30 | | Female | 105 | Y | AZA→ Rituximab→ FK506→ MMF→ FK506 | 4.5 | 1:100 | 15.89509 | 1.0 | 45% | 9 |
| 5 | 52 | | Male | 80 | Y | AZA→ FK506 | 4.5 | 1:10 | 12.41358 | 1.0 | 43% | 9 |
| 6 | 55 | | Female | 54 | Y | AZA→ MMF | 4.0 | 1:10 | 19.87432 | 1.0 | 57% | 9 |
| 7 | 62 | | Female | 240 | Y | FK506 | 5.5 | 1:32 | 22.7282 | 1.0 | 20% | 9 |
| 8 | 37 | | Female | 120 | Y | MMF | 7.0 | 1:100 | 17.13584 | 1.0 | 46% | 9 |
| 9 | 37 | | Female | 30 | Y | FK506 | 2.5 | 1:1000 | 22.65957 | 1.0 | 43% | 9 |
| 10 | 67 | | Female | 48 | Y →discontinued for intolerance | MTX→ AZA→ FK506 | 4.0 | 1:32 | 18.04612 | 1.0 | 52% | 9 |
| 11 | 47 | | Female | 101 | Y →discontinued for intolerance | MMF | 6.0 | 1:32 | 30.29883 | 1.0 | 57% | 9 |
| 12 | 47 | | Female | 64 | Y | AZA → MMF | 7.5 | 1:100 | 19.94268 | 1.0 | 37% | 9 |

Abbreviations: AZA, azathioprine; MMF, mycophenolate mofetil; MTX, methotrexate; FK506, tacrolimus; TM, transverse myelitis; ON, optic neuritis; APS, area postrema syndrome; EDSS, Expanded Disability Status Scale; CBA, cell-based assay. EDSS, Expanded Disability Status Scale.

a Percentage of infusion CD3^+^ cells expressing CAR-BCMA: CAR-BCMA was detected on the surface of CD3^+^ T cells by staining with anti-mouse IgG.

Table S2.

| Table S2. Adverse Events and CRS related to trial regimen for patients receiving CT013A cells therapy | | | | | |
| --- | --- | --- | --- | --- | --- |
| Patient | Grade 1-2 | Grade 3 | Grade 4 | CRS | Treatment for CRS management |
| 1 | Pyrexia Flu like symptoms Hypogammaglobulinemia | Leukopenia | Neutropenia Lymphocytopenia | 1, pyrexia |  |
| 2 | Nausea and Vomiting Pyrexia Hypocalcemia Hypogammaglobulinemia APTT prolonged Blood LDH increased | Anemia Diarrhea CMV infection Hypokalemia Thrombocytopenia Urinary infection | Leukopenia Neutropenia Lymphocytopenia | 1, pyrexia | 40mg methylprednisolone at D10 |
| 3 | Pyrexia Anemia Myocardial strain Hypogammaglobulinemia Urinary infection | CMV infection | Leukopenia Neutropenia Lymphocytopenia | 1, pyrexia |  |
| 4 | Pyrexia Hematuria EBV infection Hypogammaglobulinemia | Anemia Upper respiratory infection | Leukopenia Neutropenia Lymphocytopenia | 1, pyrexia |  |
| 5 | Pyrexia Anemia Leukocytosis Platelet count increased Hypogammaglobulinemia | Leukopenia CMV infection | Neutropenia Lymphocytopenia | 1, pyrexia |  |
| 6 | Pyrexia Diarrhea Flu like symptoms Neutrophilia Hypogammaglobulinemia |  | Leukopenia Neutropenia Lymphocytopenia | 1, pyrexia |  |
| 7 | Pyrexia Flu like symptoms Nausea and Vomiting Diarrhea Expectoration NT-proBNP increased AST increased Oral herpes BKV infection Hypogammaglobulinemia | CMV infection Coagulation disorder Pneumonia | Anemia Leukopenia Neutropenia Lymphocytopenia Thrombocytopenia | 1, pyrexia | 40mg methylprednisolone at D6 and D13 |
| 8 | Pyrexia Flu like symptoms Hypogammaglobulinemia | Anemia | Leukopenia Neutropenia Lymphocytopenia | 1, pyrexia |  |
| 9 | Flu like symptoms Hypogammaglobulinemia | Anemia | Leukopenia Neutropenia Lymphocytopenia | 1, pyrexia | 40mg methylprednisolone at D7 |
| 10 | Pyrexia Hypotension AST increased ALT increased Blood fibrinogen decreased NT-proBNP increased Hematuria Hypogammaglobulinemia | Anemia CMV infection Urinary infection | Leukopenia Neutropenia Lymphocytopenia Thrombocytopenia | 2, pyrexia; transient hypotension (82/54 mmHg at D5; 84/46 mmHg at D14) |  |
| 11 | Pyrexia Anemia AST increased Hypogammaglobulinemia | ALT increased | Leukopenia Neutropenia Lymphocytopenia | 1, pyrexia |  |
| 12 | Pyrexia Anemia Nausea and Vomiting Diarrhea | Leukopenia Urinary infection | Neutropenia Lymphocytopenia | 1, pyrexia |  |

Abbreviations: CMV, cytomegalovirus infection; APTT, activated partial thromboplastin time; NT-proBNP, N-terminal pro-brain natriuretic peptides; AST, aspartate aminotransferase; ALT, alanine aminotransferase; LDH, lactate dehydrogenase.

Table S3.

| Table S3. Serious Adverse Events related to trial regimen | | | | |
| --- | --- | --- | --- | --- |
|  | | 0.5×10^6^ CAR T cells/kg (N=3) | 1.0×10^6^ CAR T cells/kg (N=9) | All Patients (N=12) |
|  |  | Any Grade | | Grade 3 or higher |
| Any | | 1 (33) | 2 (22) | 3 (25) |
|  | CMV infection | 1 (33) | 2 (22) | 3 (25) |
|  | Pneumonia | 0 (0) | 1 (11) | 1 (8) |
|  | Coagulation disorder | 0 (0) | 1 (11) | 1 (8) |

Abbreviations: CMV, cytomegalovirus.

Table S4.

|  | Table S4. CAR T cells expansion in vector transgene copies | | | | |
| --- | --- | --- | --- | --- | --- |
|  |  | | 0.5×10^6^ CAR T cells/kg (N=3) | 1.0×10^6^ CAR T cells/kg (N=9) | All Patients (N=12) |
|  | T_max_ (Days) | |  | | |
|  |  | Median | 10 | 10 | 10 |
|  |  | Min, Max | 10, 10 | 10, 10 | 10, 10 |
|  | C_max_ (Copies/μg DNA) | |  | | |
|  |  | Median | 55300 | 79451 | 78025.5 |
|  |  | Min, Max | 38822, 147243 | 50600, 108945 | 38822, 147243 |
|  | AUC_0-28d_ (Days×Copies/μg DNA) | |  | | |
|  |  | Median | 329184.9 | 529081.0 | 513781.4 |
|  |  | Min, Max | 277487.7, 1500890.5 | 211368.9, 900419.1 | 211368.9, 1500890.5 |

Table S5.

| Table S5. CAR T cells expansion in CAR T-Cell percentage in CD3^+^ T lymphocytes | | | | |
| --- | --- | --- | --- | --- |
|  | | 0.5×10^6^ CAR T cells/kg (N=3) | 1.0×10^6^ CAR T cells/kg (N=9) | All Patients  (N=12) |
| T_max_ (Days) | |  | | |
|  | Median | 10 | 10 | 10 |
|  | Min, Max | 10, 14 | 10, 10 | 10, 14 |
| C_max_ (%) | |  | | |
|  | Median | 77.8 | 85.5 | 84.4 |
|  | Min, Max | 58.5, 93.5 | 48.6, 91.5 | 48.6, 93.5 |
| AUC_0-28d_ (Days×%) | |  | | |
|  | Median | 673.5 | 1250.8 | 1170.6 |
|  | Min, Max | 359.6, 1627.0 | 709.8, 1925.0 | 359.6, 1925.0 |

Data for CAR T percentage in patient 4 at day 28 post infusion was missing. Her data were not included in AUC _0-28d_.

Table S6.

| Table S6. CAR T-Cell Persistence Over Time | | | | |
| --- | --- | --- | --- | --- |
|  | Month 1 | Month 2 | Month 3 | Month 6 |
| No. of patients | 12 | 11 | 10 | 6 |
| No. (%) with detectable vector | 12 (100) | 8 (73) | 6 (60) | 1 (17) |

All 12 patients were included in the analysis.

Table S7.

| Table S7. Secondary end points for disability and quality-of-life outcomes ^a^ | | | |
| --- | --- | --- | --- |
|  | | From Baseline to Week 12 | From Baseline to Month 6 |
|  |  | All Patients (N=10) | All Patients (N=6) |
| VAS pain score^b^ | | 0.00 (-2.00,1.00) | 0.00 (-0.50,1.00) |
| FACIT-Fatigue scale^c^ | | 0.00 (-5.00,6.00) | 2.00 (-1.00,7.50) |
| SF-36 score^d^ | |  | |
|  | Physical functioning domain | 0.05 (-0.05,0.20) | 0.05 (0.00,0.38) |
|  | Role-physical domain | 0.00 (0.00,0.63) | 0.38 (0.25,1.00)^j^ |
|  | Bodily pain domain | 0.05 (-0.10,0.26) | 0.05 (-0.05,0.38) |
|  | General health domain | 0.13 (-0.13,0.30) | 0.13 (-0.20,0.40) |
|  | Vitality domain | 0.05 (-0.13,0.22) | 0.08 (-0.11,0.44) |
|  | Social role functioning domain | 0.00 (-0.31,0.31) | 0.31 (-0.06,0.75) |
|  | Role-emotional domain | 0.00 (0.00,0.00) | 0.00 (0.00,0.00) |
|  | Mental health domain | 0.06 (-0.20,0.16) | 0.02 (-0.18,0.26) |
| EQ-5D index score^e^ | | 0.04 (-0.05,0.11) | 0.07 (-0.04,0.32) |
| EQ-5D Visual Analogue Scale^f^ | | 10.00 (0.00,15.00)^h^ | 10.00 (-17.50,35.00) |
| Modified Rankin Scale^g^ | | -0.50 (-1.00,0.00)^i^ | -1.00 (-1.50,0.00) |

^a^ Changes from baseline are shown as median (95% CI).

^b^ Scores on the visual analogue scale (VAS) score for pain (on a scale from 0 to 100, with higher scores indicating more pain).

^c^ Scores on the Functional Assessment of Chronic Illness Therapy–Fatigue (FACIT-F) score (on a scale from 0 to 52, with higher scores indicating better condition or less fatigue).

^d^ Scores on the 36-item Short Form Health Survey (SF-36; eight sections with scores transformed to 0 to 100, with lower scores indicating greater disability).

^e^ Scores on the EuroQol-5 Dimensions (EQ-5D) index scores (scored on a scale from −0.109 to 1, with higher scores indicating a better health state).

^f^ Scores on the EQ-5D-VAS (on a scale from 0 to 100, with higher scores indicating better condition).

^g^ Scores on the modified Rankin scale (scored from 0 [no symptoms] to 6 [death]).

Comparison was performed by Wilcoxon matched-pairs signed rank test. ^h^ p = 0.04; ^i^ p = 0.03; ^j^ p = 0.03.

Table S8.

| Table S8. Anti-drug antibody (ADA) detected over time | | | | | | | | | | | | | |
| --- | --- | --- | --- | --- | --- | --- | --- | --- | --- | --- | --- | --- | --- |
| Time post infusion | ADA detection assay | | | | | | | | | | | | Total |
|  | Patient 1 | Patient 2 | Patient 3 | Patient 4 | Patient 5 | Patient 6 | Patient 7 | Patient 8 | Patient 9 | Patient 10 | Patient 11 | Patient 12 |  |
| Baseline | Pos | Neg | Neg | Neg | Neg | Neg | Neg | Neg | Neg | Neg | Neg | Neg | 1 |
| Month 1 | Neg | Neg | Neg | Neg | Neg | Neg | Neg | Neg | Neg | Neg | Neg | Neg | 0 |
| Month 3 | Pos | Neg | Neg | Neg | Neg | Neg | Neg | Neg | Neg | Neg |  |  | 1 |
| Month 6 | Pos | Pos | Neg | Neg | Pos | Neg |  |  |  |  |  |  | 3 |
| Month 9 | Pos | Pos |  |  |  |  |  |  |  |  |  |  | 2 |
| Month 10 | Pos^a^ |  |  |  |  |  |  |  |  |  |  |  | 1 |
| Month 12 | Pos |  |  |  |  |  |  |  |  |  |  |  | 1 |
| Total | 6 | 2 | 0 | 0 | 1 | 0 | 0 | 0 | 0 | 0 | 0 | 0 | 9 |

Abbreviations: Pos, positive; Neg, negative

Table S9.

| Table S9. Measures for Sjögren’s syndrome | | | | | | | | | | | | | | | | |
| --- | --- | --- | --- | --- | --- | --- | --- | --- | --- | --- | --- | --- | --- | --- | --- | --- |
| Patient’s No. |  | Serological changes | | | | Exocrine gland function | | | | | | ESSPRI | | | | ESSDAI |
|  |  | SSA (U/ml) | IgG (g/dl) | C3 (mg/dl) | C4 (mg/dl) | Salivary flow rate (ml/min) | | Tear Breakup Time (s) | | Schirmer’s test results (mm/5min) | | Dryness | Fatigue | Pain | Total |  |
|  |  |  |  |  |  | Unsti-mulated | Stimu-lated | OD | OS | OD | OS |  |  |  |  |  |
| 9 | Baseline | 28.04 | 12.9 | 0.74 | 0.23 | 0.416 | 1.409 | 2 | 2 | 2 | 3 | 8 | 3 | 0 | 11 | 5 |
|  | D84 | ＜2.00 | 3.2 | 0.77 | 0.29 | 0.660 | 0.825 | 6 | 5 | 4 | 4 | 2 | 1 | 0 | 3 | 4 |
| 10 | Baseline | 261.69 | 10.4 | 0.53 | 0.11 | 0.003 | 0.090 | 2 | 2 | 2 | 2 | 8 | 7 | 2 | 17 | 11 |
|  | D84 | 2.29 | 5.98 | 0.75 | 0.18 | 0.220 | 0.283 | 4 | 4 | 2 | 2 | 5 | 3 | 0 | 8 | 8 |

ESSPRI denotes EULAR Sjögren's Syndrome patient reported index. ESSDAI denotes EULAR Sjögren's syndrome disease activity index.

SSA denotes anti-SSA antibodies. IgG denotes immunoglobulin G. C3 denotes complement C3. C4 denotes complement C4.

OD denotes right eye. OS denotes left eye.
