# Supplementary figures and images for: Anti-BCMA CAR T-cell therapy CT103A in relapsed or refractory AQP4-IgG seropositive neuromyelitis optica spectrum disorders: phase 1 trial interim results
Source: Signal Transduct Target Ther. 2023 Jan 4;8:5. doi: 10.1038/s41392-022-01278-3 (PMC9810610; doi:10.1038/s41392-022-01278-3)

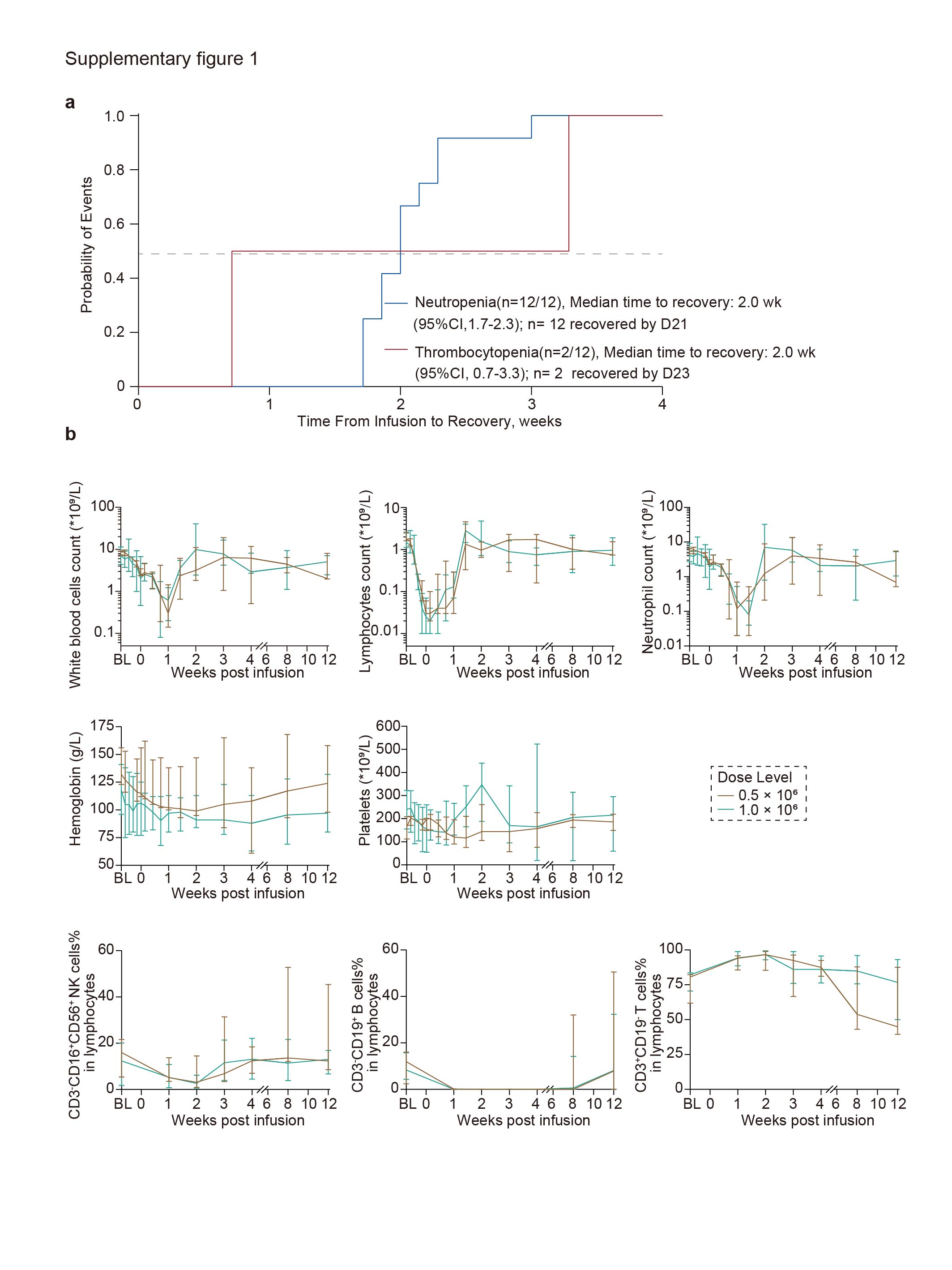

Supplement: Supplementary file 2 — supplemantary figure 1 [file 41392_2022_1278_MOESM2_ESM.jpg]

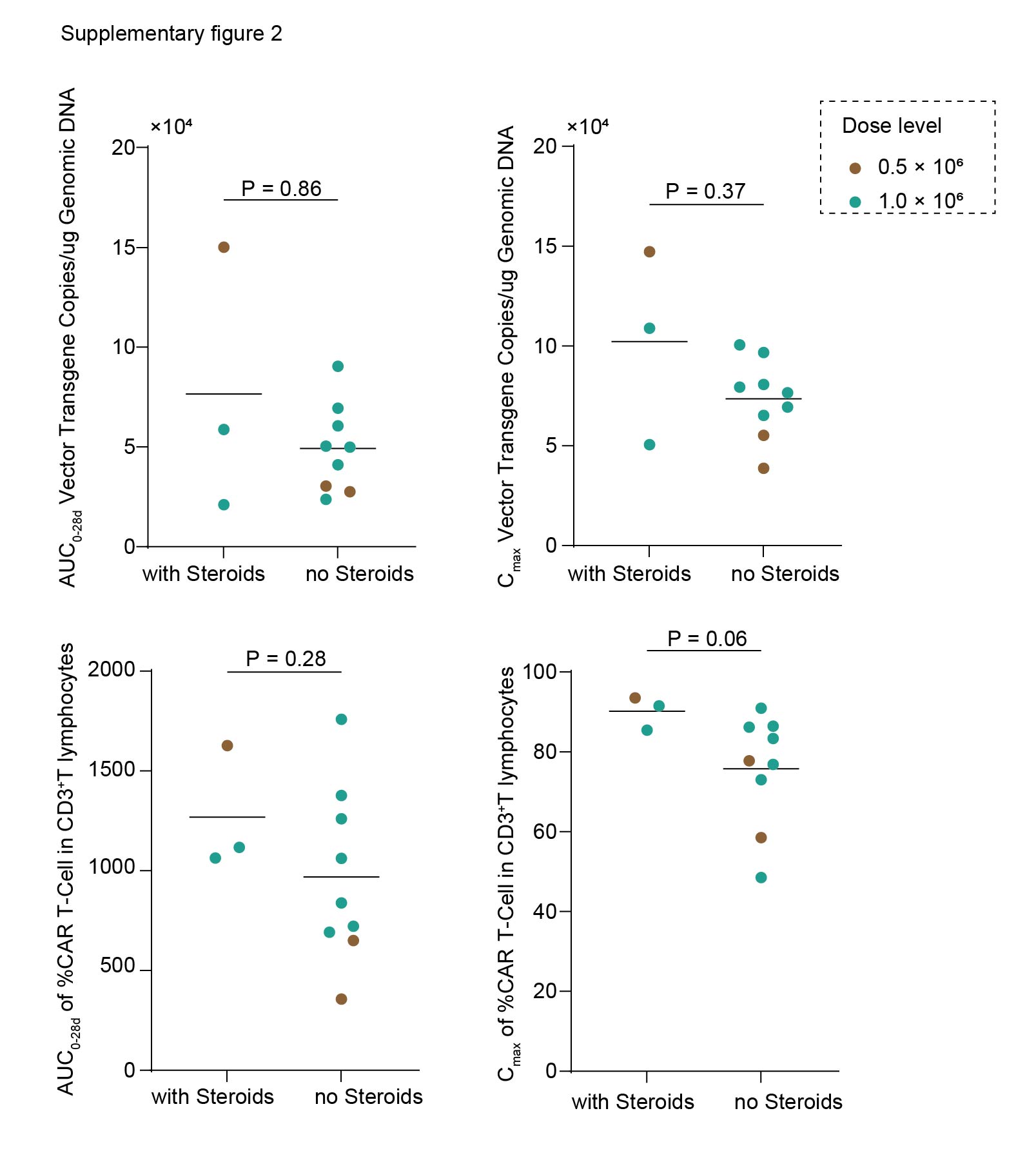

Supplement: Supplementary file 3 — supplemantary figure 2 [file 41392_2022_1278_MOESM3_ESM.jpg]

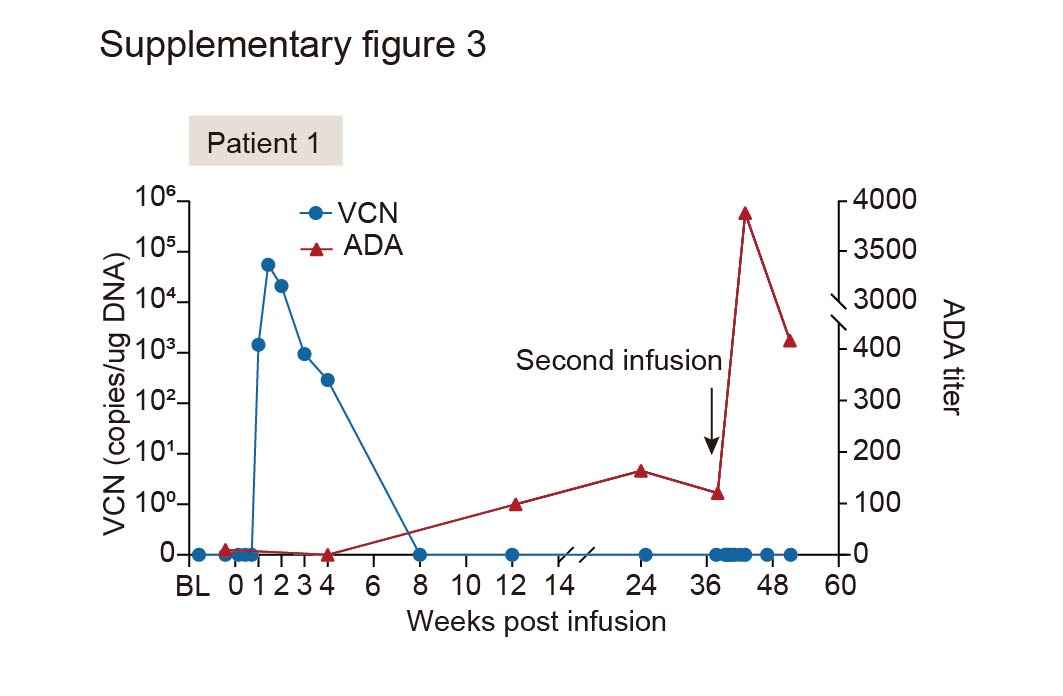

Supplement: Supplementary file 4 — supplemantary figure 3 [file 41392_2022_1278_MOESM4_ESM.jpg]

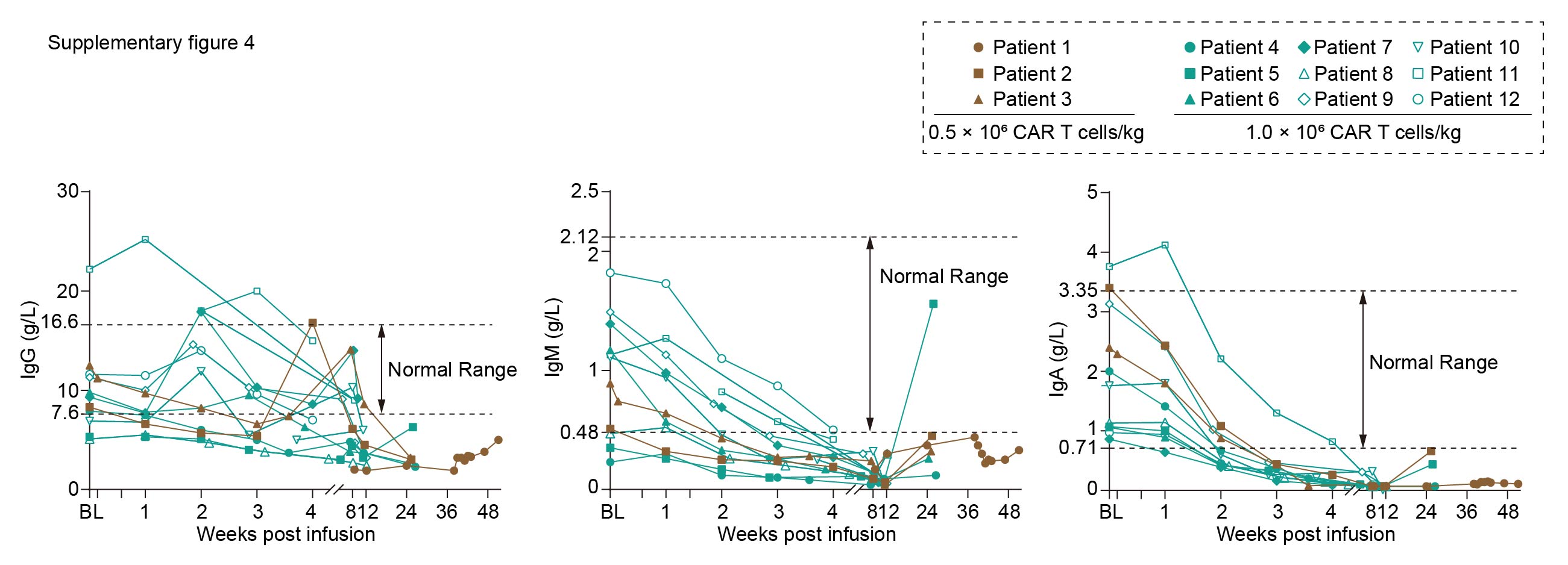

Supplement: Supplementary file 5 — supplemantary figure 4 [file 41392_2022_1278_MOESM5_ESM.jpg]

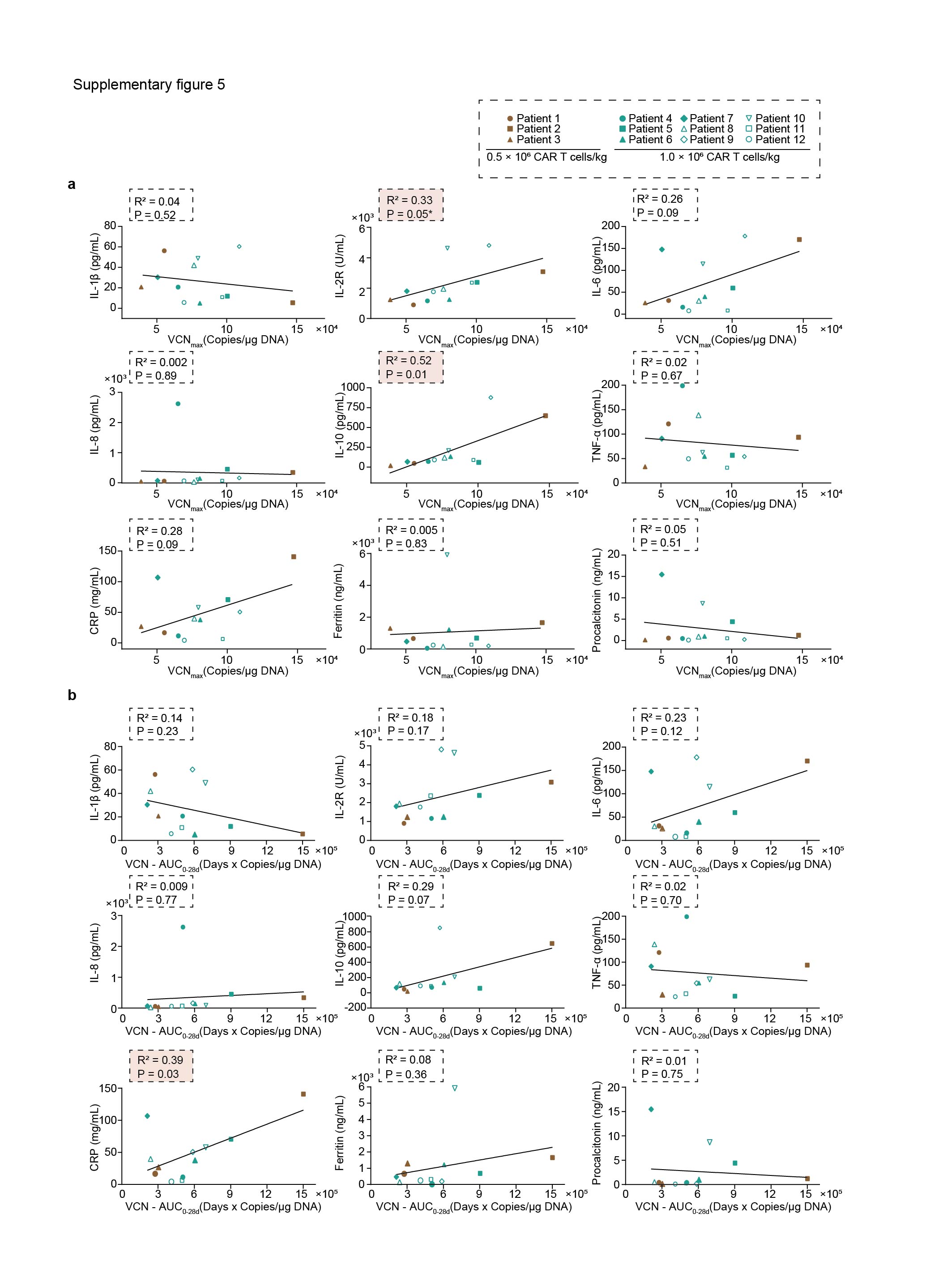

Supplement: Supplementary file 6 — supplemantary figure 5 [file 41392_2022_1278_MOESM6_ESM.jpg]

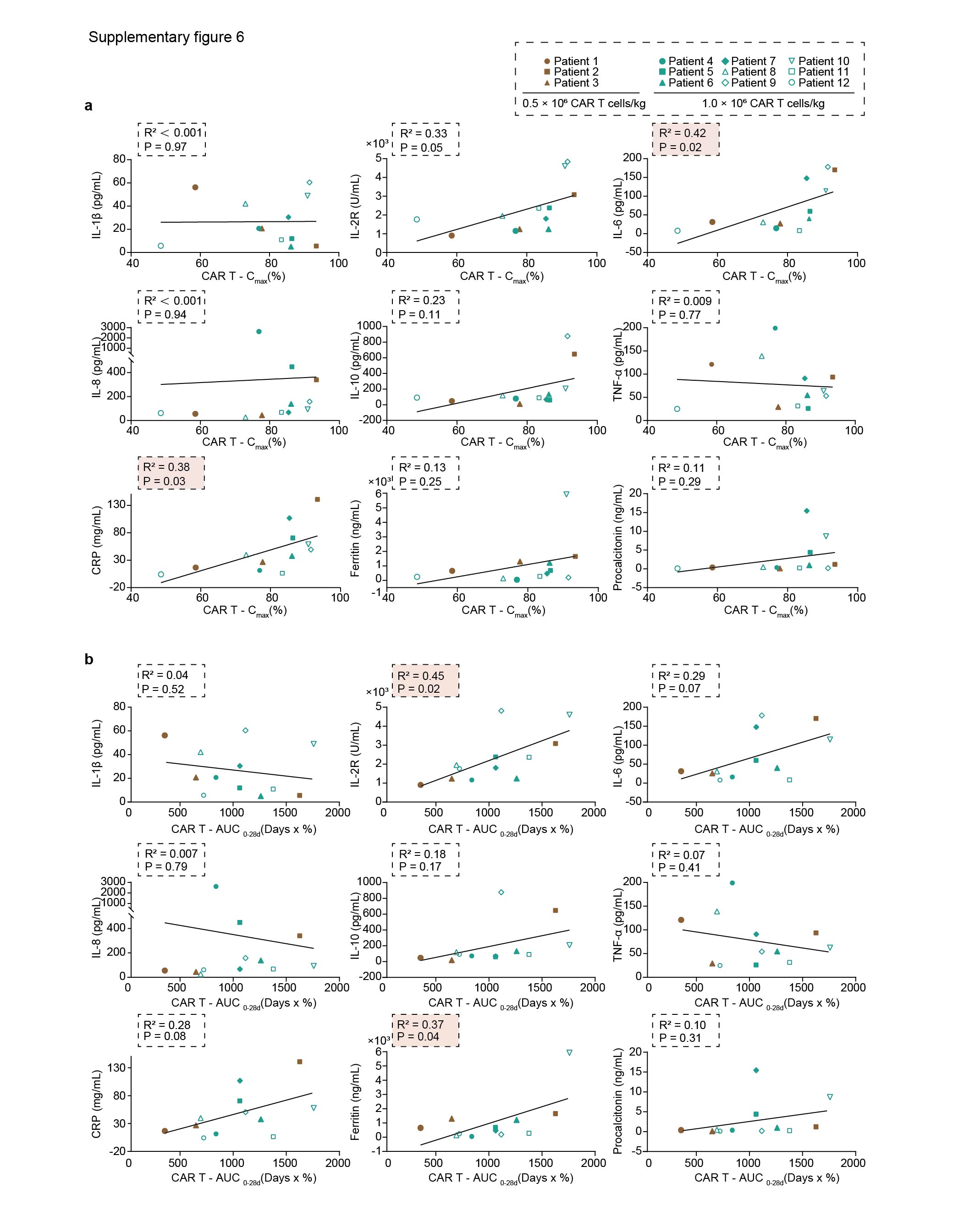

Supplement: Supplementary file 7 — supplemantary figure 6 [file 41392_2022_1278_MOESM7_ESM.jpg]

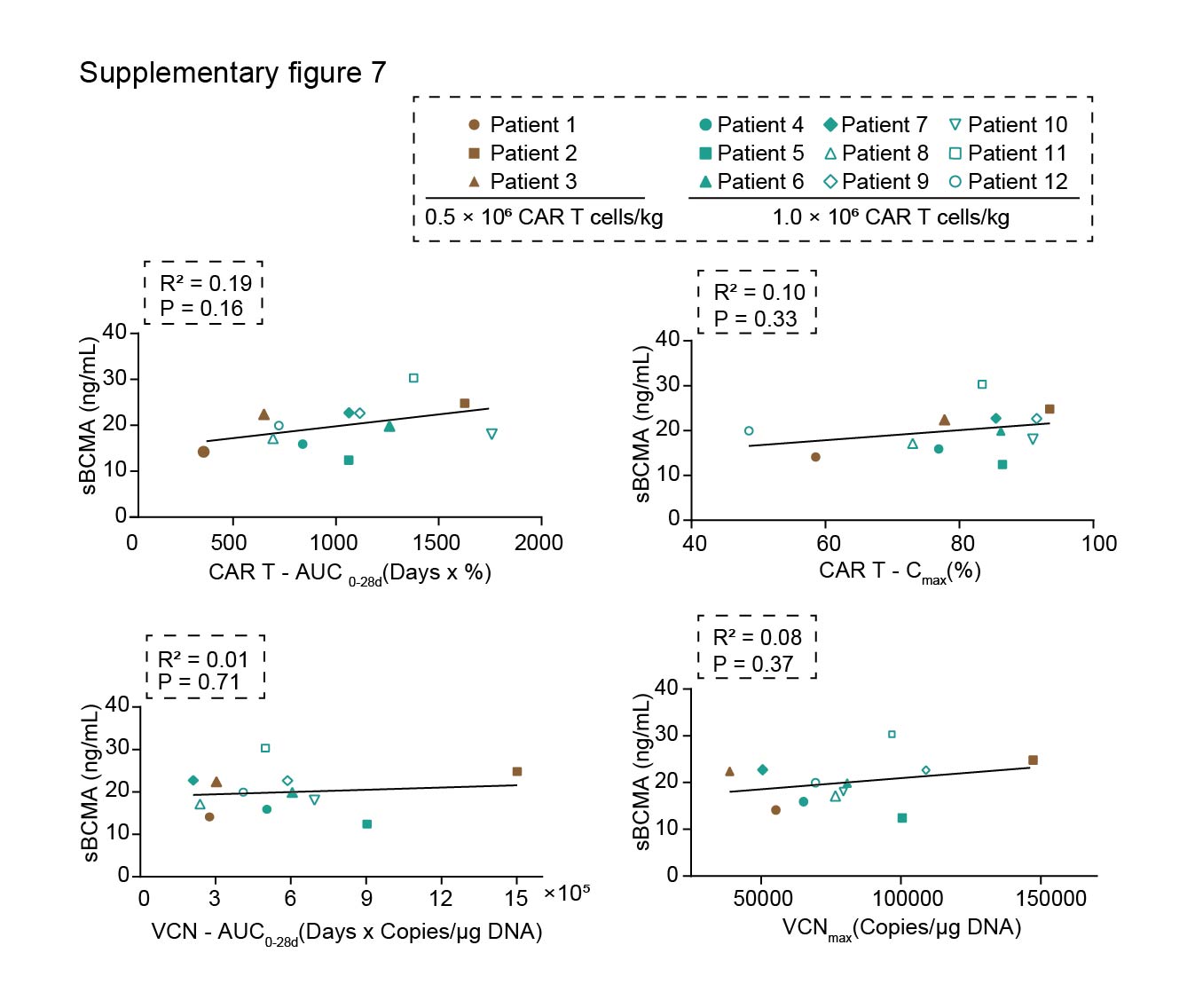

Supplement: Supplementary file 8 — supplemantary figure 7 [file 41392_2022_1278_MOESM8_ESM.jpg]
