# Supplementary material for: Anti-BCMA CAR T-cell therapy CT103A in relapsed or refractory AQP4-IgG seropositive neuromyelitis optica spectrum disorders: phase 1 trial interim results
Source: Signal Transduct Target Ther. 2023 Jan 4;8:5. doi: 10.1038/s41392-022-01278-3 (PMC9810610; doi:10.1038/s41392-022-01278-3)
Supplement: Supplementary file 9 — supplemantary protocol [file 41392_2022_1278_MOESM9_ESM.docx]

**An open -label, exploratory clinical study to evaluate the safety and efficacy of CT103A cells in relapsed/refractory antibody - associated idiopathic inflammatory diseases of the nervous system (CARTinNS)**

**Protocol number:** CNCT103AI1003

**ClinicalTrials.gov:** NCT04561557

**Version number:** 4.1

**Version Date:** November 16, 2021

**Protocol Amendments:**

Protocol version 2.0, dated 22 May 2020-ORIGINAL

(Protocol version 1.0 was reviewed by the Ethics committee, and required significant modifications before reconsideration; Protocol version 2.0 was the first protocol approved and released to the public.)

Protocol version 3.0, dated 18 September 2020 (Prior to 1st patient infused)

Protocol version 4.0, dated 24 August 2021

Protocol version 4.1, dated 16 November 2021

**Principal Investigator:** Professor Wang Wei

**Investigator:** Tongji Hospital Affiliated to Tongji Medical College, Huazhong University of Science and Technology

CAR-T preparation unit: Nanjing IASO Biotherapeutics Co., Ltd.

Study sponsor: Nanjing IASO Biotherapeutics Co., Ltd.

| Confidential Information  All of the information contained in this program is confidential and belongs to Nanjing IASO Biotherapeutics Co., Ltd. Except for the investigators listed in this plan, any third party shall not copy or disseminate the relevant information of this document without obtaining the written permission of Nanjing IASO (referring to signing the cooperation agreement). |
| --- |

**Content**

[1 Study Objectives 4](#_Toc102295028)

[2 Study design 5](#_Toc102295029)

[2.1 Study design 5](#_Toc102295030)

[2.1.1 Overall design 5](#_Toc102295031)

[2.1.2 Dose-Limiting Toxicity (DLT) 6](#_Toc102295032)

[2.1.3 Maximum tolerated dose (MTD) 6](#_Toc102295033)

[3 Subject population 7](#_Toc102295034)

[3.1 Subject and sample size estimates 7](#_Toc102295035)

[3.2 Inclusion criteria 7](#_Toc102295036)

[3.3 Exclusion criteria 7](#_Toc102295037)

[3.4 Lymphodeletion and CAR-T infusion criteria 8](#_Toc102295038)

[4 Experimental cell products and treatment options 10](#_Toc102295039)

[4.1 Cell product information 10](#_Toc102295040)

[4.2 Preparation of cell products 10](#_Toc102295041)

[4.3 Storage conditions 10](#_Toc102295042)

[4.4 Packaging and labeling of cell products 10](#_Toc102295043)

[4.5 Lymphodepletion 10](#_Toc102295044)

[4.6 CT103A cell infusion 10](#_Toc102295045)

[4.7 CRS and recommendations for its management 11](#_Toc102295046)

[5 Study procedures 13](#_Toc102295047)

[5.1 Study flow table 13](#_Toc102295048)

[5.2 Arrangement 31](#_Toc102295049)

[5.3 Extended follow-up period Vn (every 6 months ± 14 days) 31](#_Toc102295050)

[6 Trial evaluation and adverse events 34](#_Toc102295051)

[6.1 Evaluation 34](#_Toc102295052)

[6.1.1 Main evaluation 34](#_Toc102295053)

[6.1.2 Secondary evaluation 34](#_Toc102295054)

[6.2 Adverse Events and Serious Adverse Events 35](#_Toc102295055)

[6.2.1 Definition 35](#_Toc102295056)

[6.2.2 Evaluation of Adverse Events 36](#_Toc102295057)

[6.2.3 Follow-up for AEs 37](#_Toc102295058)

[6.2.4 Outcome of AEs 38](#_Toc102295059)

[6.3 Pregnancy report 38](#_Toc102295060)

[6.4 Safety assessment 39](#_Toc102295061)

[6.5 Clinical laboratory tests 39](#_Toc102295062)

[6.6 Effectiveness Assessment 43](#_Toc102295063)

[6.7 Pharmacokinetic evaluation 43](#_Toc102295064)

[6.7.1 Pharmacokinetic blood sampling 43](#_Toc102295065)

[6.7.2 Pharmacokinetic blood sample processing 43](#_Toc102295066)

[6.7.3 Pharmacokinetic blood test 43](#_Toc102295067)

[6.8 Exploratory evaluation 43](#_Toc102295068)

[6.8.1 NMOSD disease history 43](#_Toc102295069)

[6.8.2 Disease recurrence/onset assessment 44](#_Toc102295070)

[6.8.3 Imaging assessment 44](#_Toc102295071)

[6.8.4 Functional status assessment 44](#_Toc102295072)

[6.8.5 Visual function assessment 44](#_Toc102295073)

[6.8.6 Quality of life assessment 45](#_Toc102295074)

[6.8.7 Other exploratory research blood sample collection and testing 45](#_Toc102295075)

[6.8.8 Exploratory study blood sample processing 45](#_Toc102295076)

[7 Biological sample analysis 45](#_Toc102295077)

[8 Statistical Analysis 46](#_Toc102295078)

[8.1 General principles 46](#_Toc102295079)

[8.2 Population analysis 46](#_Toc102295080)

[8.3 Content analysis 47](#_Toc102295081)

[8.3.1 Case distribution 47](#_Toc102295082)

[8.3.2 Protocol deviation 47](#_Toc102295083)

[8.3.3 Demographic data and baseline analysis 47](#_Toc102295084)

[8.3.4 Treatment compliance and drug exposure analysis 47](#_Toc102295085)

[8.3.5 Concomitant medication and non-drug concomitant therapy 47](#_Toc102295086)

[8.4 Safety Analysis 47](#_Toc102295087)

[8.4.1 AE analysis 47](#_Toc102295088)

[8.4.2 Laboratory test 48](#_Toc102295089)

[8.4.3 Vital signs 48](#_Toc102295090)

[8.4.4 Physical examination 48](#_Toc102295091)

[8.5 Efficacy analysis 48](#_Toc102295092)

[8.6 Pharmacokinetic (PK) Analysis 48](#_Toc102295093)

[8.7 Exploratory Analysis 48](#_Toc102295094)

[9 Ethics and Informed Consent 50](#_Toc102295095)

[9.1 Laws and Regulations 50](#_Toc102295096)

[9.2 Ethics Committee 50](#_Toc102295097)

[9.3 Informed consent 50](#_Toc102295098)

[10 Clinical trial data management and storage 50](#_Toc102295099)

[10.1 Requirement of investigator to fill in data 50](#_Toc102295100)

[10.2 EDC data management 51](#_Toc102295101)

[10.3 External data transfer 51](#_Toc102295102)

[10.4 Source file 51](#_Toc102295103)

[References 52](#_Toc102295104)

# Study Objectives

In patients with relapsed/refractory AQP4 antibody-associated NMOSD, the aims of this study are as follows:

**Primary objectives:**

1. To evaluate the safety and tolerability of CT103A cells in the treatment of subjects with relapsed/refractory neuromyelitis optica spectrum disease (NMOSD)
2. To explore the recommended doses for expansion (RDEs) of CT103A cells in the treatment of subjects with relapsed/refractory NMOSD, providing evidence for subsequent studies.

**Secondary objectives:**

1. To preliminarily evaluate the efficacy of CT103A cells in the treatment of subjects with relapsed/refractory NMOSD.
2. To determine the pharmacokinetic (PK) characteristics of CT103A cells in subjects with relapsed/refractory NMOSD.

**Exploratory objectives:**

1. To observe the efficacy of CT103A cells in the treatment of subjects with relapsed/refractory NMOSD.
2. Pharmacodynamic (PD) characterization of CT103A cells in subjects with relapsed/refractory NMOSD.
3. To examine changes in inflammatory factors in subjects after CT103A infusion.
4. To assess immunogenicity of CT103A in humans.
5. To analyze shifts in lymphocyte subgroups after CT103A infusion.
6. To monitor the level of replicative lentivirus in vivo.

# Study design

## Study design

### Overall design

This study is an exploratory clinical study in patients with relapsed/refractory antibody - associated idiopathic inflammatory disease of the nervous system , including a dose-escalation phase and a dose-expansion phase.

The dose-escalation phase trial is planned to enroll 6-12 subjects with refractory AQP4 antibody-positive NMOSD.

In order to maximize the safety of patients after receiving treatment, CT103A cells with an initial dose of 0.5×10 ^6^ CAR-T cells/Kg are selected for infusion. Tolerance and safety, the preliminary efficacy, and PK, PD indicators will be observed.

If DLT does not occur in the first 3 subjects in the 0.5×10 ^6^ CAR-T cells/Kg dose group, and curative effect is observed in ≥1 subject, the investigator can use the obtained preliminary efficacy information, PK/PD parameters and safety data to make one of the following choices: (1) Continue the enrollment in the 0.5×10 ^6^ CAR-T cells/Kg dose group, and stop when 6 DLT-evaluable cases are completed; (2) Stop enrolling in the 0.5×10 ^6^ CAR-T cells/Kg dose group, and convert to the 1×10 ^6^ CAR-T cells/Kg dose group for DLT evaluation.

If DLT does not occur in the first 3 subjects in the 0.5×10 ^6^ CAR-T cells/Kg dose group, but curative effect is observed in none of the subjects, the dose will be escalated to 1×10 ^6^ CAR-T cells/Kg dose for DLT evaluation.

If 1 case of DLT is observed in the first 3 cases in the 0.5×10 ^6^ CAR-T cells/Kg dose group, continue to enroll up to 6 DLT-evaluable cases in this dose group.

If there are 2 cases of DLT in the 0.5×10 ^6^ CAR-T cells/Kg dose group, the investigator should decide whether to switch to a lower dose group of 0.25×10 ^6^ CAR-T cells/Kg dose, based on the preliminarily attained efficacy and PK/PD parameters.

The first 3 subjects in each dose group need to be enrolled one by one, and the time interval between infusions of every two subjects is at least 2 weeks. If the subject has a good tolerance and benefits from the treatment of this regimen (disease remission after CT103A cell infusion), and the investigator determines that the infusion may benefit the subject; the subject can receive re-lymphodepletion treatment and re-infusion of CT103A according to the condition of infusion of CT103A defined in the protocol. If the condition deteriorates, or unacceptable toxicity occurs entailing a change in the treatment regimen, or the investigator deems that the subject is not suitable for continuation, or the subject requests a withdrawal, he/she should have a discharge visit within 14 days after withdrawal and before starting a new treatment regimen.

By analyzing the safety, efficacy, PK and PD data of CT103A treatment in the dose-escalation phase, we can determine the recommended dose (RDE) for the dose-expansion phase study. Based on the above analysis results, we may modify the study protocol and conduct a dose-expansion phase study on the RDE dose group of CT103A under the premit of obtaining the approval of the ethics committee.

If the trial enters the dose expansion phase, it is expected to enroll 6-30 subjects with NMOSD, and considered the inclusion of other relapsed/refractory antibody-associated neurological idiopathic diseases with similar pathogenesis and treatment mechanism of NMOSD. Each newly included disease will have its own treatment group, which must be authorized by the ethics committee prior to enrollment. If the preliminary efficacy evaluation results of some treatment groups in the dose expansion stage suggest that there is value and significance for further exploration, the number of enrolled cases will continue to be expanded under the premit of the approval of the ethics committee, so that it conforms to the statistics standards for the calculation of the efficacy evaluation sample size of the indication population.

### Dose-Limiting Toxicity (DLT)

CTCAE of Grade 3-5 (CTCAE version 5.0) newly occurring within 28 days after CT103A infusion, which are related to CT103A treatment (definitely related, likely related, possibly related). EXCEPT FOR the following events:

- Grade ≥ 3 CRS and neurotoxicity that can be relieved to Grade ≤ 2 within 3 days after best supportive care (refer to the 2019 ASTCT recommended CRS and neurotoxicity grading criteria)
- Hematological toxicity:
- Grade 3 NEUT count decrease of any duration or Grade 4 NEUT count decrease lasting ＜ 28 days
- Grade 3 anaemia of any duration or Grade 4 anaemia lasting ＜ 28 days
- Grade 3 platelet count decrease of any duration or Grade 4 platelet count decrease lasting ＜ 28 days
- Decreased peripheral blood counts other than the above
- Grade ≥ 3 nonhematologic toxicities that resolve to Grade ≤ 2 within 7 days

### Maximum tolerated dose (MTD)

MTD is defined as the highest dose at which DLT occurs in ≤ 33% of subjects (at least 6 subjects in this dose group).

If ≥ 2 DLTs occurs at the same dose level, enrolment at the current dose should be suspended, and this dose level is defined as MAD.

# Subject population

## Subject and sample size estimates

CT103A is a CAR-T cell product targeting BCMA, a member of the tumor necrosis factor (TNF) superfamily of proteins primarily expressed on plasma cells and some mature B cells. Based on the key role of plasma cells and the production of pathogenic AQP4-IgG in NMOSD, this trial selects patients with relapsed/refractory AQP4-IgG seropositive NMOSD.

The experimental product CT103A has already obtained the clinical results of the first-in-human phase I clinical trial in relapsed/refractory plasma cell tumors, but the current trial is the first time when CT103A is used in the treatment of patients with relapsed/refractory antibodies-associated idiopathic inflammatory diseases of the nervous system. 6-12 subjects with relapsed/refractory NMOSD are selected for the dose escalation phase trial.

## Inclusion criteria

Subjects must meet all of the following criteria for inclusion:

1. Male or female, aged 18 to 75 (including 18 and 75 years old);
2. Remission of all acute toxicities associated with previous treatment to baseline or ≤ Grade 1 (NCI-CTCAE v5.0, except for toxicities deemed by the investigator to be no safety risk to the subject);
3. AQP4-IgG–seropositive relapsed/refractory NMOSD previously diagnosed by the 2015 International NMO Diagnostic Panel (IPND) NMOSD diagnostic criteria, meeting the following requirements:
   - With at least one immunosuppressant for more than one year with suboptimal symptom control;
   - At least 2 recurrences within 12 months prior to enrolment, or at least 3 recurrences within 24 months prior to enrolment plus at least 1 recurrence within the last 12 months;
4. The subject's organ function is good at the time of screening, and the laboratory test data meets the following criteria:
   - Blood routine: absolute neutrophil count ≥ 2.0 × 10 ^9^ /L (or greater than the lower limit of the normal range of the research center laboratory);
   - Liver function: serum total bilirubin ≤ 2 times the upper limit of the normal range (ULN), AST and ALT ≤ 2 times the ULN;
   - Renal function: CrCl ≥ 60 ml/min /1.73 m ^2^ (calculated according to Cockcroft-Gault formula);
   - Electrolytes: serum potassium ≥ 3.0 mmol/L, serum calcium ≥ 2.0 mmol/L, serum magnesium ≥ 0.5 mmol/L;
   - Coagulation function : fibrinogen ≥ 1.0g/L, activated partial thromboplastin time (APTT) ≤ ULN+10s, prothrombin time (PT) ≤ ULN+3s;
5. Oxygen saturation ＞ 91% at rest;
6. Left ventricular ejection fraction (LVEF) ≥ 50 %;
7. According to the investigator's judgment, the expected survival period of the subjects is ≥ 12 weeks;
8. Consent to use contraceptive methods such as double-barrier techniques, condoms, oral or injectable contraceptives, or intrauterine devices, during the study period and for one year following the final study medication;
9. Prior to the study's commencement, subjects must provide written informed consent.

## Exclusion criteria

1. Inability to collect non-mobilized peripheral blood mononuclear cells for CAR-T cell production;
2. History of autoimmune haemolytic disease;
3. History of solid organ transplantation;
4. Treatment with alemtuzumab within 6 months before apheresis, or fludarabine or cladribine within 3 months before apheresis;
5. Papilloma vacuolar virus nucleic acid test positive;
6. History of unresolved malignancy within 2 years (the following conditions can be excluded from the 2-year limit: non-melanoma skin cancer, completely resected stage I tumour with low probability of recurrence, limited-stage prostate cancer after treatment, biopsy-proven cervical in situ carcinoma, or PAP smear showing squamous intraepithelial lesions);
7. Hepatitis B surface antigen (HBsAg) or hepatitis B core antibody (HBcAb) positive and peripheral blood hepatitis B virus (HBV) DNA test value > 100 IU/mL ; Hepatitis C virus (HCV) antibody positive and peripheral blood hepatitis C virus (HCV) RNA positive; Human immunodeficiency virus (HIV) antibody positive; Cytomegalovirus (CMV) DNA positive; syphilis positive;
8. Recognized primary immunodeficiency (congenital or acquired);
9. Serious heart disease: including but not limited to unstable angina, myocardial infarction (within 6 months prior to screening), congestive heart failure (New York Heart Association [NYHA] classification Grade ≥ III), severe arrhythmia;
10. Cerebrovascular accident, including transient ischemic attack or stroke, occurred within 6 months before enrolment;
11. Major surgery or surgical treatment for any reason within 4 weeks prior to enrolment;
12. Any serious and/or uncontrolled comorbidities that the investigator believes may interfere with the study assessment;
13. Previous treatment:
    1. History of thymectomy within 12 months before CT103A cell infusion;
    2. Therapeutic doses of corticosteroids (defined as > 40 mg/day of prednisone or similar) within 10 days prior to PBMC apheresis, except that physiological replacement therapy, topical use, and inhaled corticosteroids are permitted;
    3. Immunosuppressive agents within 7 days before PBMC apheresis;
    4. Rituximab treatment within 5 months prior to signing ICF;
    5. Gamma globulin treatment within 1 week prior to enrollment;
    6. Plasma exchange or double filtration within 1 week before enrolment;
14. History of mental disorder or a history of psychotropic substance abuse without withdrawal;
15. Habitual drinking of grapefruit juice or excessive tea, coffee and/or caffeinated beverages and inability to withdraw during the trial;
16. Allergic constitutionor a history of severe allergies;
17. Pregnant or breastfeeding women;
18. There were cases that were deemed unsuitable for enrolment by the investigators.

## Lymphodeletion and CAR-T infusion criteria

Reassessments are essential before pretreatment of lymphodepletion and before CAR-T cell infusion, respectively. Those who meet the following conditions cannot be infused:

1. Hematology: neutrophil ＜ 2 × 10 ^9^ /L, platelet count ＜ 50 × 10 ^9^ L;
2. Oxygen is required to maintain blood oxygen saturation ≥ 91%;
3. Creatinine clearance ＜ 50 mL/min;
4. Subjects with the following conditions, including but not limited to: new-onset arrhythmias that cannot be controlled with medication, hypotension requiring vasopressors, bacterial, fungal, or viral infections requiring intravenous antibiotics (except those with antibiotics used for infection prevention);
5. Subjects who need to take therapeutic doses of corticosteroids (except for physiological replacement therapy) or immunosuppressive agents after the start of lymphodepletion or during the study period;
6. For any reason, the infusion of cells is delayed for at least 7 days after the lymphodepletion (at this time, the investigator needs to evaluate whether it is necessary to perform the lymphodepletion again);
7. There are situations in which other investigators consider it unsuitable for lymphodepletion or cell infusion.

# Experimental cell products and treatment options

## Cell product information

CT103A cells are genetically modified autologous T cells that express single-chain BCMA antibody extracellularly and transmit signals through a signaling region linked in tandem with intracellular 4-1BB and CD3ζ. CT103A needs to be refrigerated and can be treated in 1 bag.

## Preparation of cell products

Quality control of cell products is done in the Reindeer production laboratory. CT103A is prepared from peripheral blood mononuclear cells of subjects. Mononuclear cells enriched for T cells are then transduced with lentivirus expressing a CAR targeting BCMA and activated with magnetic beads coated with anti-CD3/CD28 antibodies.

## Storage conditions

Cryopreservation bags (10-50ml capacity) containing CT103A cells need to be kept in the gas phase storage space of a liquid nitrogen tank, and stored and transported at a temperature not higher than -130 degrees Celsius, no more than 7 days.

## Packaging and labeling of cell products

This cell product has three layers of packaging. Cryopreservation bags are individually packaged aseptically to prevent contamination during the freezing process. The outer packaging is a stainless-steel storage box, which is convenient for storage and transportation. Freezer bags all contain label bags that can be inserted with written product information to ensure label integrity during the freeze-thaw process.

The labeling of cell products will be carried out in accordance with the guiding principles of GCP and other relevant regulations, clearly marked "for clinical research use only", and will include key information such as cell product name, subject identification, product batch number, and cell number.

## Lymphodepletion

2-4 days before CT103A cell infusion

The recommended lymphodepletion programs are as follows:

Cyclophosphamide 500mg/m ^2^ IV over 30 minutes, once daily for 3 consecutive days; Fludarabine 30mg/m ^2^ IV over 30 minutes (immediately after cyclophosphamide infusion is completed), once a day for 3 consecutive days.

## CT103A cell infusion

After lymphodepletion, according to the assigned dose group, CT103A cells of the specified dose will be used for infusion. Intravenous administration of CT103A injection, , is completely given within 30 minutes, 1 bag each time.

Indications for readministration of CT103A:

The expansion of CT103A in the patient is monitored, and the cells are reinfused according to the following indications:

1. At least 8 weeks have passed since the last cell infusion;

2. No DLT or Grade ≥4 CRS ;

3. At least a certain degree of remission of the disease should be observed after the first and previous cell infusion (refer to the evaluation of effectiveness) or disease progression or acute attack after remission;

4. The vector copy number (VCN) decreases below 10^3^ copies/μg DNA within 90 days;

5. The number of CT103A cells produced in the past is sufficient for re-infusion, or PBMC collection can be reperformed.

For subjects who are going to be re-infused, inclusion and exclusion criteria need to be rechecked. PBMC collection can be reperformed if the recheck work is confirmed appropriate; if there are CT103A cells stored within the validity period, PBMC collection can be exempted. Before lymphodepletion and reinfusion, it is necessary to confirm that it meets the standard criteria of lymphodepletion and cell reinfusion. Then, the cells are infused again according to the procedure and dosage of the first cell infusion. To maximize subjects’ benefits, efficacy and tolerance after infusion (Grade ≥ 3 AEs related to cell infusion occur after the previous CT103A infusion) are the key to determine whether to down-regulate or up-regulate the dose level of cell reinfusion.

Whether a subject needs to undergo reinfusion or not, will be decided by the investigator after comprehensive judgment and discussion with the sponsor, based on the overall condition of the subject.

## CRS and recommendations for its management

CRS is due to the release of inflammatory cytokines such as TNF-α, IL-1 , IL-6 , IL-12 , IFN-α , IFN-β , IFN-γ , MCP-1 and IL-8, leading to syndrome of symptoms such as fever, nausea, headache, tachycardia, hypotension, rash, and shortness of breath. Systemic symptoms such as fever, fatigue, anorexia, and myalgia are usually clinical manifestations. Other organs and systems can also be affected, including the cardiovascular system, respiratory system, skin, gastrointestinal tract, liver, kidney, blood system, and nervous system.

Multiple standards for CRS classification exist at home and abroad. The standard (CRS grading standard recommended by ASTCT in 2019) in this study uses fever, blood pressure level, and blood oxygen saturation for CRS grading. Mild CRS can be treated with symptomatic and supportive treatment, which can often resolve spontaneously. The treatment of moderate-to-severe CRS focuses on early identification and timely treatment. In the early stage, manifestations of moderate-to-severe CRS are sensitive to tocilizumab and glucocorticoids, but in the late stage they become resistant.

- If there is no relief after treatment, or IL-6 ＞ 500-1000 pg/mL within 3 days, or IL-6 rises sharply within one day, high fever persists, and symptoms continue to deteriorate, regardless of CRS grade, it is recommended to use tocilizumab and glucocorticoids at the same time. Glucocorticoids could be used until symptoms are relieved.
- If the subject has ferritin ＞ 20,000 ug/L, persistent high fever, and continued aggravation of symptoms and signs, regardless of the CRS grade, it is recommended to use tocilizumab and glucocorticoids at the same time, and glucocorticoids can be used until symptoms ease.
- For severe leak syndrome and cardiac insufficiency, continuous hemofiltration (CRRT) and/or plasma exchange is recommended as an effective treatment.
- If the patient's IL-6 declines and then sharply rises again during treatment, it is recommended to consider severe infections such as sepsis.
- Vasopressor, mechanical ventilation, and plasma exchange are all recommended effective in treatment to severe CRS.

# Study procedures

Signed and dated informed consent form must be obtained from the subject (or their guardian) prior to any screening steps. For all subjects considered for admission to the trial, the investigator/designee will document the degree of compliance with the inclusion criteria on the screening enrollment form. For subjects considered for possible inclusion in this trial, if they were ultimately ineligible for inclusion, the screening inclusion form should also be completed and the reasons for ineligibility recorded. The screening inclusion form for subjects who do not meet the trial inclusion criteria must be kept at the trial center.

## Study flow table

See next page:

| **Visit content** | | **Screening period** | **PBMC collection ^1^** | **Lymphodepletion^2^** | | | **DLT observation period for CT103A infusion** | | | | | | | | | **Long-term follow-up period** | | | | | | | |  |  |
| --- | --- | --- | --- | --- | --- | --- | --- | --- | --- | --- | --- | --- | --- | --- | --- | --- | --- | --- | --- | --- | --- | --- | --- | --- | --- |
|  |  | **V1** | **V2** | **V3** | | **V4** | **V5** | | | | | | | | | **V6** | **V7** | **V8-9** | **V10** | **V11-13** | **V14** | **Vn** | **Out-of-group visit** |  |  |
|  |  | **D-42 ~ D-20** | | **D-12 ~ D-5** | **D-4 ~ D-2** | **D**  **-1** | **D0** | **D1** | **D**  **3** | **D**  **5** | **D**  **7** | **D**  **10** | **D**  **14±1 days** | **D**  **21  ±1 day** | **D**  **28  ± 3 days** | **D**  **56  ± 7 days** | **D**  **84  ± 7 days** | **1 time/every 3 months ± 14 days** | **D**  **365**  **±14 days** | **1 time/every 3 months ± 14 days** | **D**  **730  ± 14 days** | **1 time/every 6 months  ± 14 days** | **Indication to change long-term treatment regimens^21^** |  |  |
| **Sign ICF** | | **X** |  |  |  |  |  |  |  |  |  |  |  |  |  |  |  |  |  |  |  |  |  |  |  |
| **Inclusion/Exclusion Criteria** | | **X** |  |  |  |  |  |  |  |  |  |  |  |  |  |  |  |  |  |  |  |  |  |  |  |
| **Demographic information** | | **X** |  |  |  |  |  |  |  |  |  |  |  |  |  |  |  |  |  |  |  |  |  |  |  |
| **Disease diagnosis and treatment history ^3^** | | **X** |  |  |  |  |  |  |  |  |  |  |  |  |  |  |  |  |  |  |  |  |  |  |  |
| **Medical history ^4^** | | **X** |  |  |  |  |  |  |  |  |  |  |  |  |  |  |  |  |  |  |  |  |  |  |  |
| **Past treatment ^5^** | | **X** |  |  |  |  |  |  |  |  |  |  |  |  |  |  |  |  |  |  |  |  |  |  |  |
| **EDSS Score** | |  |  | **X** |  | **X** |  |  |  |  |  |  | **X** |  | **X** | **X** | **X** | **X** | **X** | **X** | **X** | **X** | **X** |  |  |
| **Serum AQP4 antibody titers ^6^** | | **X** |  | **X** |  |  |  |  | **X** |  | **X** | **X** | **X** | **X** | **X** | **X** | **X** | **X** | **X** | **X** | **X** | **X** | **X** |  |  |
| **Cerebrospinal fluid routine, biochemistry, immunoglobulin, oligoclonal band, IgG index and inflammatory factors** | |  |  | **X** |  |  |  |  |  |  |  |  |  |  | **X** |  | **X** | **X ^20^** | **X** | **X ^20^** |  |  |  |  |  |
| **MRI ^7^** | |  |  | **X** |  |  |  |  |  |  |  |  |  |  | **X** |  | **X** | **X** | **X** | **X ^20^** | **X** | **X** |  |  |  |
| **VEP and visual acuity ^8^** | |  |  | **X** |  |  |  |  |  |  |  |  |  |  | **X** |  | **X** | **X** | **X** | **X** | **X** | **X** |  |  |  |
| **OCT** | |  |  | **X** |  |  |  |  |  |  |  |  |  |  |  |  | **X** | **X** | **X** | **X** | **X** | **X** |  |  |  |
| **DTI** | |  |  | **X** |  |  |  |  |  |  |  |  |  |  |  |  | **X** | **X** | **X** | **X** | **X** | **X** |  |  |  |
| **Vital signs ^9^** | | **X** | **X** | **X** | **X** | **X** | **X** | **X** | **X** | **X** | **X** | **X** | **X** | **X** | **X** | **X** | **X** | **X** | **X** | **X** | **X** | **X** | **X** |  |  |
| **Weight, height, body surface area ^10^** | | **X** | **X** | **X** |  |  | **X** |  |  |  |  |  |  |  |  |  |  |  |  |  |  |  |  |  |  |
| **Physical examination** | | **X** | **X** | **X** | **X** | **X** | **X** | **X** | **X** | **X** | **X** | **X** | **X** | **X** | **X** | **X** | **X** | **X** | **X** | **X** | **X** | **X** | **X** |  |  |
| **Blood oxygen saturation** | | **X** |  |  |  | **X** | **X** | **X** | **X** | **X** | **X** | **X** | **X** | **X** | **X** |  |  |  |  |  |  |  |  |  |  |
| **Infectious Disease ^11^** | | **X** |  |  |  |  |  |  |  |  |  |  |  |  |  |  |  |  |  |  |  |  |  |  |  |
| **Pregnancy** | | **X** |  | **X** |  |  |  |  |  |  |  |  |  |  |  |  |  |  |  |  |  |  | **X** |  |  |
| **Blood routine** | | **X** | **X** | **X** | **X** | **X** | **X** | **X** | **X** | **X** | **X** | **X** | **X** | **X** | **X** | **X** | **X** | **X** | **X** | **X** | **X** | **X** | **X** |  |  |
| **Blood biochemistry ^12^** | | **X** |  | **X** |  | **X** |  |  | **X** |  | **X** |  | **X** | **X** | **X** | **X** | **X** | **X** | **X** | **X** | **X** | **X** | **X** |  |  |
| **Coagulation** | | **X** |  | **X** |  | **X** |  |  | **X** |  | **X** | **X** | **X** | **X** | **X** | **X** | **X** | **X** | **X** | **X** | **X** | **X** | **X** |  |  |
| **Urine routine** | | **X** |  | **X** |  |  |  |  |  |  |  |  | **X** |  | **X** | **X** | **X** | **X** | **X** | **X** | **X** | **X** | **X** |  |  |
| **Stool for routine and** **occult blood** | | **X** |  |  |  |  |  |  |  |  |  |  |  |  |  |  |  |  |  |  |  |  |  |  |  |
| **Lymphocyte subsets and function detection** | |  |  | **X** |  | **X** |  |  |  |  | **X** |  |  | **X** | **X** | **X** | **X** | **X** | **X** |  |  |  | **X** |  |  |
| **ECG** | | **X** |  | **X** |  |  |  |  |  |  |  |  |  |  | **X** | **X** | **X** | **X** | **X** | **X** | **X** | **X** | **X** |  |  |
| **echocardiography** | | **X** |  | **X** |  |  |  |  |  |  |  |  |  |  |  |  |  |  |  |  |  |  | **X** |  |  |
| **exploratory study** | **inflammatory factor ^13^** |  |  | **X** |  | **X** |  | **X** | **X** | **X** | **X** | **X** | **X** | **X** | **X** | **X** | **X** |  |  |  |  |  | **X** |  |  |
|  | **Human anti-CAR antibody detection** |  |  | **X** |  |  |  |  |  |  |  |  |  |  | **X** |  | **X** | **X** | **X** | **X** | **X** | **X** | **X** |  |  |
|  | **Replicating Lentivirus Detection** |  |  | **X** |  |  |  |  |  |  |  |  |  |  |  |  | **X** | **X** | **X** | **X** | **X** | X | **X** |  |  |
| **PK ^14^ _** | **Peripheral blood CAR-T detection** |  |  | **X** |  |  |  | **X** | **X** | **X** | **X** | **X** | **X** | **X** | **X** | **X** | **X** | **X** | **X** | **X** | **X** | **X** | **X** |  |  |
|  | **Lentivirus copy number detection** |  |  | **X** |  |  |  | **X** | **X** | **X** | **X** | **X** | **X** | **X** | **X** | **X** | **X** | **X** | **X** | **X** | **X** | **X** | **X** |  |  |
| **Peripheral blood free BCMA** | |  |  | **X** |  |  |  |  |  |  | **X** |  | **X** | **X** | **X** | **X** | **X** | **X** | **X** | **X** | **X** | **X** | **X** |  |  |
| **Peripheral blood mononuclear cell collection** | |  | **X** |  |  |  |  |  |  |  |  |  |  |  |  |  |  |  |  |  |  |  |  |  |  |
| **Lymphodepletion** | |  |  |  | **X** |  |  |  |  |  |  |  |  |  |  |  |  |  |  |  |  |  |  |  |  |
| **CT103A infusion** | |  |  |  |  |  | **X** |  |  |  |  |  |  |  |  |  |  |  |  |  |  |  |  |  |  |
| **CRS Assessment ^15^** | |  |  |  |  |  | **X** | **X** | **X** | **X** | **X** | **X** | **X** | **X** | **X** |  |  |  |  |  |  |  |  |  |  |
| **ICANS Assessment ^16^** | |  |  |  |  |  | **X** | **X** | **X** | **X** | **X** | **X** | **X** | **X** | **X** |  |  |  |  |  |  |  |  |  |  |
| **SF-36, VAS pain, mRS, EQ-5D, FACIT-fatigue** | |  |  | **X** |  |  |  |  |  |  |  |  |  |  | **X** | **X** | **X** | **X** | **X** | **X** | **X** | **X** | **X** | **X** | **X** |
| **Adverse Events ^17^** | | **X** | **X** | **X** | **X** | **X** | **X** | **X** | **X** | **X** | **X** | **X** | **X** | **X** | **X** | **X** | **X** | **X** | **X** | **X** | **X** | **X** | **X** |  |  |
| **Serious Adverse Events ^18^** | | **X** | **X** | **X** | **X** | **X** | **X** | **X** | **X** | **X** | **X** | **X** | **X** | **X** | **X** | **X** | **X** | **X** | **X** | **X** | **X** | **X** | **X** |  |  |
| **Concomitant therapy ^19^** | | **X** | **X** | **X** | **X** | **X** | **X** | **X** | **X** | **X** | **X** | **X** | **X** | **X** | **X** | **X** | **X** | **X** | **X** | **X** | **X** |  | **X** |  |  |

Notes:

1. During the screening period, after the subjects complete the corresponding visit content inspection according to the visit schedule, they will be assessed by the investigator to meet the inclusion criteria and will receive the PBMC collection process according to the standard operating procedure (SOP) for PBMC collection. If the screening period and the cell preparation period (-42~-20d) are due to the subject's delayed lymphodepletion and/or cell infusion, the time from the subject's signature to the time of infusion exceeds 6 weeks specified in the protocol, which will be ignored.
2. Examinations completed within 1 week before the start of the lymphodepletion will be regarded as the baseline, and the lymphodepletion is performed on -4d ~- 2d.
3. Inquire the subject in detail about the diagnosis history of the study disease and collect diagnostic support materials (reports and medical records containing the diagnosis), and the patient's treatment process for the study disease (if detailed information is available, the name of the treatment should be included as much as possible, and the treatment method includes drug administration, drug route and dosage, start and end time of treatment, disease remission after treatment, time of disease deterioration and recurrence).
4. Diseases other than the study disease within 5 years before the entry to the signing of the informed consent form, have been cured or recovered to a state of no clinical significance during the screening period, and will be recorded as the past medical history.
5. Drug or non-drug treatment (including nutritional supplements such as vitamins, vehicles or solvents with therapeutic effects) from 4 weeks before enrollment to the signing of informed consent will be recorded as the past therapy history.
6. Serum AQP4 antibody titers are measured only once at the time of signing ICF and at baseline within one week prior to pretreatment.
7. Subjects need to accept MRI examinations of the optic nerve and central nervous system. MRI scans and enhancement of the head, cervical spine, thoracic spine, lumbar spine, and optic nerve are required. Test results within 3 days are acceptable.
8. VEP needs to be recorded including the latency time.
9. Axillary temperature should be measured 3-4 times a day within 14 days after CT103A cell infusion (6-8 hours interval is recommended). Measure axillary temperature twice a day for 14-28 days. As long as the body temperature is higher than 37.8 degrees Celsius, out-of-hospital subjects should contact the investigator immediately. If the body temperature is greater than 37.8 degrees Celsius, and the CRP is > 20mg/dl or rises rapidly, the subject needs to be hospitalized immediately until the body temperature drops to normal for 24 hours. Subjects should not use non-steroidal anti-inflammatory drugs by themselves within 28 days after CT103A cell infusion, so as not to affect the monitoring of body temperature. Elevated body temperature is sometimes the only precursor to fatal CRS.
10. During the screening period, weight, height, and body surface area are measured (calculation formula: body surface area = SQRT (height cm* weight Kg/3600)) , and only body weight is measured at subsequent visits.
11. Infectious disease screening: blood- FC050_JC virus nucleic acid detection, HbsAg or HBcAb, peripheral blood hepatitis B virus, hepatitis C virus specific antibody (HCVAb) and human immunodeficiency virus specific antibody (HIVAb).
12. Biochemical blood samples should be collected on an empty stomach. The tests include K , Na , Mg , Cl , Ca , CREA , UREA , URIC , TCHO , TG , LDL-C , HDL-C , TP , ALB , TBIL , DBIL , ALT , AST , ALP , LDH , GLU , CK , CK-MB.
13. Inflammatory factors include ferritin, IL-6, C -reactive protein, procalcitonin, of which IL-6 does not need to be tested alone on D14 , D28 and D84 (at the listed visit point " Lymphocyte subsets and function testing" includes IL -6).
14. PK blood collection is performed 5 to 12 days before CT103A cell infusion (time window: before lymphodepletion), 1 day after infusion (time window ± 8 hours), 3 days (time window ± 8 hours), 5 days (time window ± 12 hours), 7 days (time window ± 12 hours), 10 days (time window ± 12 hours), 14 days (time window ± 1 day), 21 days (time window ± 1 day), 28 days (time window ± 3 days), 56 days (time window ± 7 days), 84 days (time window ± 7 days), and every 3 months (time window ± 14 days) after 84 days , until that CAR-Thas monitored the peak value for 2 consecutive times without detection of VCN by ddPCR, or disease deteriorates, or till 2 years after group withdrawal or infusion. 1 day after infusion represents 24 hours from the time of infusion, 3 days after infusion represents 72 hours from the time of infusion, and so on. It should be noted that blood routine sampling should be performed at the same time as PK sampling every time, and the time interval between them should not exceed 24 hours.
15. Hospitalization is essential within 2 weeks after CT103A infusion, and CRS assessment is performed at least twice a day.
16. ICANS assessments should be performed twice a day within 2 weeks after CT103A infusion.
17. Laboratory examination results, or clinically significant aggravation of the aforementioned events from the signing of ICF should be recorded as adverse events, and should be judged whether to be related to PBMC collection, lymphodepletion, or CT103A cell infusion.
18. SAE, from the signing of ICF until 2 years after cell infusion, CT103A-related tumors and positive RCL tests that occurred after CT103A infusion should be reported in the form of SAE.
19. Medications (including nutritional supplements such as vitamins, and therapeutic vehicles or solvents) and non-pharmacological treatments should be recorded as concomitant treatments since the signing of the ICF. It should be continued until 2 years after CT103A cell infusion (if no disease deteriorates or relapse occurs at 24 months) or at the out-of-group visit. If the disease deteriorates or relapses within 6 months after the CT103A cell infusion, the cells should be collected as much as possible 6 months after the infusion under the premise of the subject's consent.
20. The cerebrospinal fluid-related inflammatory factors included NFL, GFAP and sTREM2 should be deteceted at only 6 months and 12 months after CT103A cells infusion.
21. In addition to short-term treatment (including corticosteroid treatment, immunosuppressant, gamma globulin infusion, etc.) during the acute attack of the disease, if the investigator determines that the long-term treatment regimen needs to be changed, the subject needs to complete the group visit within 14 days after the judgment before switching to a new treatment regimen.

## Arrangement

CT103A cells are infused as day 0

## Extended follow-up period Vn (every 6 months ± 14 days)

Subjects will enter the extended follow-up period after they complete the 2 -year long-term observation and follow-up. They will be followed up every 6 months until that the indication of CT103A cell reinfusion again, or of changing the long-term treatment plan present. If so, the subjects needed to complete the out-of-group visit before switching to a new treatment regimen.

1. EDSS Score
2. Serum AQP4 antibody titer detection
3. MRI, OCT and DTI
4. VEP and visual acuity
5. Vital signs, physical examination
6. Clinical laboratory test: blood routine (collected at the same time as PK blood sampling, the longest time interval should not exceed 24 hours), blood biochemistry, coagulation, urine test.
7. PK detection: Detection items include peripheral blood CAR-T detection, and lentivirus copy number detection.
8. Exploratory indicators: human anti-CAR antibody level detection, and lentivirus replication detection.
9. Peripheral blood free BCMA
10. ECG
11. SF-36 assessment, VAS pain assessment, mRS assessment, EQ-5D assessment, FACIT-fatigue assessment
12. Replicative lentivirus testing once a year until 15 years after cell infusion
13. Adverse event
14. Serious adverse event

**Re-infusion**

According to the CT103A reinfusion conditions stipulated in the protocol, subjects who plan to undergo reinfusion of CT103A need to be rechecked on the input and discharge criteria. If the input and discharge standards are met, PBMC collection can be conducted. If there are CT103A cells stored within the validity period, PBMC collection can be exempted. Subjects who are planned to be reinfused should be re-treated with lymphodepletion and CT103A re-infusion according to the flow chart, and the reinfusion day will be used as D0 for subsequent follow-up visits.

**Out-of-group visit**

For subjects who withdraw from the study earlier, an out-of-study visit should be conducted as soon as possible, unless the subject refuses it. In addition to short-term treatments (including corticosteroid treatment, immunosuppressive agents, gamma globulin infusion, etc.) during the acute attack of the disease, if there is a clinical indication to change the long-term treatment plan, the subject needs to complete the out-of-group visit before changing treatment regimen.

If a new treatment regimen has started before the completion of the out-of-group visit, unless the patient withdraws ICF, all items of the out-of-group visit should be completed as soon as possible within the time window, and the out-of-hospital diagnosis of disease deterioration and treatment process should be recorded in detail in chronological order. The efficacy evaluation of the out-of-group visit does not need to be repeated within 4 weeks from the last examination; the RCL test of the withdrawal visit does not need to be repeated within 3 months from the last examination after the infusion; other examinations are required to be repeated when they have been tested more than 1 week from the last inspection to withdrawal.

1. EDSS Score
2. Serum AQP4 antibody titer detection
3. Vital signs, physical examination
4. Serum pregnancy
5. Clinical laboratory test: Blood routine (collected at the same time as PK blood sampling, the longest time interval should not exceed 24 hours), blood biochemistry, coagulation, urine test.
6. Lymphocyte subsets and function detection
7. Exploratory indicators: Inflammatory Factors
8. PK detection: Detection items include peripheral blood CAR-T detection, and lentivirus copy number detection.
9. Exploratory indicators: human anti-CAR antibody level detection, and lentivirus replication detection.
10. Peripheral blood free BCMA
11. ECG, echocardiography
12. SF-36 assessment, VAS pain assessment, mRS assessment, EQ-5D assessment, FACIT-fatigue assessment
13. Adverse event
14. Serious adverse event
15. Concomitant treatment

# Trial evaluation and adverse events

## Evaluation

### Main evaluation

**Safety evaluation:**

- - Types and incidence of DLT
  - Types and incidence of AEs (except CRS and ICANS assessed according to the criteria of NCI-CTCAE v5.0); incidence and grade of CRS and I CANS; evaluation of the changes of vital signs and physical examination results before and after treatment; assessment of the changes of laboratory test results such as hematology, liver and kidney function before and after treatment; estimation of the changes of cardiac function such as ECG, echocardiography and other complementary test results before and after treatment.

### Secondary evaluation

**Efficacy evaluation:**

- - Changes in serum AQP4 antibody levels within 3 months.

**Pharmacokinetics (PK blood collection case):**

- - The copy number of BCMA CAR gene in peripheral blood after administration (VCN copies/ μg DNA);
  - The concentration of BCMA CAR-T cells in peripheral blood after administration detected by flow cytometry (cells/mL);

**Exploratory Evaluation Metrics:**

- - Time to first relapse: time from cell infusion to first NMOSD relapse (days);
  - Annualized relapse rate (ARR): The number of NMOSD relapses in subjects after cell infusion divided by the observation time (years);
  - Number of total cumulative active MRI lesions;
  - EDSS scores before and after treatment;
  - Modified Rankin scale scores;
  - VEP and visual acuity;
  - Annualized hospitalization frequency: the number of disease-related hospitalizations (overnights) in subjects after cell infusion divided by the time of observation (years);
  - Health Status Questionnaire: SF -36 Scale Scores;
  - Health Index Scale: EQ-5D Scores;
  - VAS pain score;
  - CAR-T-related serum cytokines such as ferritin, CRP, IL-6, and procalcitonin;
  - Serum AQP4 antibody levels (degree of decline, area under the curve AUC _0-90d_);
  - The concentration of free BCMA in peripheral blood before and after treatment (ng/mL);
  - Human anti-CAR antibody level;
  - Replicative lentivirus copy number;
  - Lymphocyte subsets.

## Adverse Events and Serious Adverse Events

### Definition

#### Adverse event (AE)

Adverse events refer to all adverse medical events that occur after subjects receive the investigational drug, which can be manifested as symptoms, signs, diseases, or abnormal laboratory tests, but are not necessarily causally related to the investigational drug.

Any events included here are that new or that have worsened in severity or frequency from baseline, including abnormal laboratory findings.

AE does not include:

1. Medical or surgical procedures (e.g., surgery, endoscopy, tooth extraction, fluid infusion), for which the disease leading to these procedures should be reported as an AE;
2. Existing or detected prior to the signing of ICF, but without an aggravating disease or condition, including abnormal laboratory results;
3. Anticipated progression of study disease and/or expected progression of symptoms and signs of study disease, unless the severity or frequency of occurrence is higher than expected.

#### Serious Adverse Event (SAE)

Serious adverse events refer to the following adverse medical events (at any dose) that occur after subjects receive the investigational drug:

1. Death.
2. Life-threatening events: The event leads the subject to be under threat of death at the time of it. Adverse events that could lead to death in theoretically more severe cases are not included.
3. Hospitalization or extended hospitalization.
4. Permanent or severe disability or loss of function:

The term disability refers to the severe impairment of an individual's ability to perform normal life functions. This definition excludes events of relatively minor clinical significance, such as uncomplicated headache, nausea, vomiting, diarrhea, influenza, and accidental trauma (eg, ankle sprain), which may interfere with daily functioning but do not result in significant loss of function.

1. Congenital anomalies or birth defects in offspring.
2. Other important medical events:

In certain circumstances, medical and scientific judgment must be used to determine whether to expedite reporting, such as important medical events that may not be immediately life-threatening, resulting in death or hospitalization, but where medical measures are required to prevent one of these situations from occurring, which is also considered serious in general.

### Evaluation of Adverse Events

#### Severity assessment of adverse events

All AEs, except CRS and neurotoxicity, are assessed for severity using the NCI CTCAE 5.0 Adverse Event Severity Rating Scale.

Table 1 Adverse Event Severity Rating Scale

| **Level** | **Severity** |
| --- | --- |
| Level 1 | Mild; asymptomatic or mild; clinical or diagnostic only; no treatment required. |
| Level 2 | Moderate; minor, topical, or non-invasive treatment required; age-appropriate instrumental ADL ^*^. |
| Level 3 | Severe or medically significant but not immediately life-threatening; resulting in or prolonged hospitalization; disability; limited self-care activities of daily living ^**^. |
| Level 4 | Life-threatening consequences; urgent medical attention required. |
| Level 5 | AE-related deaths. |

** Instrumental activities of daily living include cooking, buying clothes, using the phone, managing money, etc.*

*** Self-care activities of daily living refer to bathing, dressing and undressing, eating, washing, taking medicine, etc., without being bedridden.*

Grading of CRS and neurotoxicity will be recorded according to ASTCT Consensus Grading for Cytokine Release Syndrome and Neurologic Toxicity Associated with Immune Effector Cells published by ASTCT in 2019.

#### Correlation assessment of adverse events to study drug

The investigator should assess whether the adverse event is related to the study drug based on his or her knowledge of the subject and the context in which the event occurs, as well as any potential possible causes. The causal relationship between AE and study drug is as follows:

1. Certainly related: There is a reasonable time relationship between the drug use and the occurrence of AEs; the reaction disappears or rapidly decreases and improves after drug withdrawal (i.e., de-challenge positive); the AE of re-drug reoccurs (i.e., rechallenge positive), and may be significantly aggravated; at the same time, it is supported by the investigator's manual or literature; and other confounding factors such as the underlying diseases have been excluded.
2. Probably related: There is no history of repeated medication, and the rest is the same as "definitely related"; or although there is a combination of medication, the possibility of AEs caused by the combination of medication can be basically ruled out.
3. Possibly related: There is a close relationship between drug use and the occurrence time of AEs, and there is supporting evidence in the literature; however, there are more than one drugs that cause AEs, or the factor of the original disease progression cannot be ruled out.
4. Possibly irrelevant: AEs are not closely related to the duration of medication; the clinical manifestations do not match the known AEs of the drug; the development of the underlying disease may also have similar clinical manifestations.
5. Certainly irrelevant: No medication; or no correlation between medication use and the occurrence time of the AE; or there exists another clear cause for the adverse event.

Certainly related, likely related, and possibly related are all classified as drug adverse reactions.

### Follow-up for AEs

Investigators should follow up for each AE. If some SAEs and related AEs still present at the end of the AE/SAE collection and recording, these SAEs/AEs should be followed-up to:

1. Resolution or recovery to baseline status or stability;
2. The investigator believes that there will be no further improvement;
3. The subject begins a new treatment regimen for the study disease;
4. When more information was not available (subject died, or subject refused to provide more information, or there was evidence that subject was lost to follow-up despite best efforts).

The time from AEs to recovery should be recorded in the source file during the study to allow verification of the original data.

For AE/SAE of Special Concern and Pregnancy Events, the sponsor or other designee may obtain additional case information by telephone, fax, email, and/or monitoring to achieve a more complete medical evaluation of these reported cases.

### Outcome of AEs

Investigators should determine the outcome of adverse events based on the outcomes of the subjects' adverse events. The outcomes of AEs are as follows:

1. Healed: Subject fully recovered from AE without any residual effects or injuries.
2. Recovery to baseline: The subject's AE returns to the value of the physical measure after entry into the trial and prior to the use of the product.
3. In improvement: Signs and symptoms associated with the event have alleviated, but not completely gone.
4. Recovered with sequelae: The subject has recovered, but with residual effects or injuries. These residual effects may be temporary but persisting at the time of reporting. If sequelae are not considered permanent, additional information will need to be provided at follow-up when events change.
5. Persistent: Signs and symptoms associated with the event are not relieved and the subject's condition remains unchanged; this AE would be persistent if the patient died due to another AE instead of this one.
6. Exacerbation: The signs and symptoms associated with the event are not alleviated and the subject's condition deteriorates.
7. Death: Only SAEs that cause death can choose "Death" as the outcome. All other AEs/SAEs present at the time of death should be reported.
8. Unknown: When subjects are lost to follow-up and investigators are unable to determine the outcome.

## Pregnancy report

To ensure the safety of the subjects, if the female subject or the female partner of the male subject becomes pregnant during the treatment of the study drug or within 1 year after the drug, the pregnancy report form needs to be completed within 24 hours of being informed. To the sponsor, at the same time, the pregnancy must be followed up to determine pregnancy outcomes (if the male subject's female partner agrees to collect information), including spontaneous or induced abortion, delivery details, congenital anomalies, or maternal or neonatal comorbidities, etc. The pregnancy report form to report will be filled out.

Spontaneous abortion, ectopic pregnancy, induced abortion due to medical and health reasons, stillbirth, neonatal SAEs (not limited to neonatal death, all congenital malformations/birth defects) are considered serious adverse events (SAEs). Along with reporting and filling the pregnancy report form, the SAE form is also required to be completed and reported. Elective abortion without complications is not considered an AE. If other SAEs occur during pregnancy, the SAE form must also be completed and reported.

## Safety assessment

Clinical safety assessments begin at the screening period by laboratory tests, measurements of vital signs, physical examination, and the subject's medical history, to detect new abnormalities and/or worsening of previous conditions. Throughout the clinical trial, medical history and physical examination, vital signs, oxygen saturation, ECG, echocardiography, laboratory tests, AE monitoring, CRS assessment, and ICANS assessment will be used for the safety assessment of this trial. DLT and MTD are determined in dose escalation studies.

## Clinical laboratory tests

Clinical laboratory tests include: blood oxygen saturation, blood routine, blood biochemistry, coagulation, cerebrospinal fluid routine, cerebrospinal fluid biochemistry, cerebrospinal fluid immunologlobulin, oligoclonal band, IgG index, urine test, serum pregnancy, stool for routine and occult blood test. In the screening period, infectious disease screening includes Hepatitis B six items, Hepatitis C virus specific antibody (HCV-Ab) and Human immunodeficiency virus specific antibody (HIV-Ab)). If laboratory findings are abnormal during the study, they will be recorded as AEs only if judged by the investigator to be clinically significant. If necessary, re-examinations may be performed to confirm the accuracy of laboratory test results.

Table 2 Blood routine test parameters

| **Blood routine (potassium EDTA test tube)** | |
| --- | --- |
| White blood cell count (WBC) | Neutrophil percentage (N%) |
| Red blood cell count (RBC) | Lymphocyte percentage (L%) |
| Platelets (PLT) | Percentage of monocytes (M%) |
| Haemoglobin (HbG) | Eosinophil percentage (EO%) |
| Neutrophil count (NEUT)  Lymphocyte count (LY) | Basophil percentage (BASO%) |

Table 3 Biochemistry test parameters

| **Blood biochemistry (serum separation tube)** | |
| --- | --- |
| Potassium (K) | Albumin (ALB) |
| Sodium (Na) | Total bilirubin (TBIL) |
| Magnesium (Mg) | Direct Bilirubin (DBIL) |
| Chlorine (Cl) | Alanine aminotransferase (ALT) |
| Calcium (Ca) | Aspartate aminotransferase (AST) |
| Creatinine (CREA) | Lactate dehydrogenase (LDH) |
| Urea (UREA) | Alkaline Phosphatase (ALP) |
| Total cholesterol (TCHO) | Glucose (GLU) |
| Triglycerides (TG) | Creatine Kinase (CK) |
| Low Density Lipoprotein (LDL-C) | Creatine kinase MB isoenzyme (CK-MB) |
| High Density Lipoprotein (HDL-C) | Uric |
| Total protein (TP) |  |

Table 4 Coagulation test parameters

| **Coagulation (sodium citrate tube)** | |
| --- | --- |
| Activated partial prothrombin time (APTT) | International Normalized Ratio (INR) |
| Prothrombin time (PT)  Thrombin time assay (TT) | Fibrinogen (FIB) |

Table 5 Urine routine test parameters

| **Urine routine (fresh urine sample)** | |
| --- | --- |
| Urinary white blood cells | Urine red blood cells |
| Urine protein | ketone bodies |
| Urine glucose | pH |

Table 6 Blood pregnancy test parameters

| **Serum Pregnancy (Serum Separator Tube)** | |
| --- | --- |
| β-HCG |  |

Table 7 Stool for routine and occult blood test parameters

| **Stool** for routine and **occult blood (fresh stool)** | |
| --- | --- |
| dung color |  |
| traits |  |
| red blood cells |  |
| leukocyte |  |
| Occult blood |  |

Table 8 Infectious disease screening

| **HBV, HCV, HIV (vacuum tube)** |
| --- |

**Blood-FC050_JC virus nucleic acid detection**

| Hepatitis B six items: hepatitis B surface antigen (HBsAg), hepatitis B surface antibody (anti-HBs), hepatitis B e antigen (HBeAg), hepatitis B e antibody (anti-HBe), hepatitis B core antibody (anti-HBc), hepatitis B virus (HBV DNA)  Hepatitis C virus specific antibody (HCV-Ab) , HCV RNA  Human Immunodeficiency Virus Specific Antibody (HIV-Ab)  Blood-FC050_JC virus nucleic acid detection |
| --- |

Table 9 Cerebrospinal fluid routine parameters

| Colour | Red blood cell count |
| --- | --- |
| Turbidity  Protein characterization | Nucleated cell count |

Table 10 Parameters of cerebrospinal fluid biochemical examination

| Glucose | CSF total protein |
| --- | --- |
| Lactate dehydrogenase  Lactic acid | CSF albumin  CSF potassium |
| CSF sodium | CSF chloride |

Table 11 Cerebrospinal fluid immunologlobulin test parameters

| CSF IgG | CSF IgM |
| --- | --- |
| CSF IgA |  |

Table 12 Cerebrospinal fluid oligoclonal band test parameters

| CSF oligoclonal band |  |
| --- | --- |
| Serum oligoclonal band |  |

## Effectiveness Assessment

**AQP4 titer in serum**

To assess the change of the serum AQP4 antibody titer of NMOSD before and after treatment, about 2ml of venous blood is collected. The specific collection time is as follows: screening period (D-42~D-20), 5 to 12 days before cell infusion (time window: no later than 1 minute before lymphodepletion), 3 days (time window ± 8 hours), 7 days (time window ± 12 hours) , 10 days (time window ± 12 hours) , 14 days (time window ± 1 day), 21 days (time window ± 1 day), 28 days (time window ± 3 days), 56 days (time window ± 3 days), 84 days (time window ± 7 days), once every 3 months (time window ± 14 days) within 2 years after 84 days.

## Pharmacokinetic evaluation

### Pharmacokinetic blood sampling

From the baseline (D-1 day), the PK test is performed at each visit until the 730th day or out of the group (day 0: just before CT103A infusion and 1 hour ± 3 minutes after the infusion, all need to be sampling). Each time 3 mL of venous blood is collected.

The specific collection time is as follows: 5 to 12 days before CT103A cell infusion (time window: no later than 1 minute before lymphodepletion), 1 day after infusion (time window ± 1 hour), 3 days (time window ± 8 hours), 5 days (time window ± 12 hours), 7 days (time window ± 12 hours), 10 days (time window ± 12 hours), 14 days (time window ± 1 day), 21 days (time window ± 1 days ), 28 days (time window ± 3 days ), 56 days (time window ± 7 days), 84 days (time window ± 7 days), once every 3 months (time window ± 14 days) within 2 years after 84 days, until CAR-T not detected by ddPCR for 2 consecutive times after the peak value of CAR-T monitoring, or disease progression, or be out-of-group, or 2 years after infusion.

### Pharmacokinetic blood sample processing

Blood samples should be handled, stored and transported in accordance with laboratory regulations.

### Pharmacokinetic blood test

After all collected specimens are sent to the laboratory, the laboratory will perform pharmacokinetic testing using validated methods.

## Exploratory evaluation

As to NMOSD, referring to EDSS score, the number of active lesions on MRI, ARR and the percentage of subjects without recurrence, the efficacy of the final treatment is comprehensively evaluated by the investigators based on the subject's EDSS score, disease-related laboratory tests before and after treatment, and neurological specialist auxiliary examinations.

### NMOSD disease history

In the screening period, the history of NMOSD disease of the subjects should be collected completely, including the time of initial diagnosis of NMOSD, the date of previous recurrence, the treatment regimen, and the calculated previous ARR (the normalized total number of cases per person-year within the 24 months before enrollment). A history of other autoimmune diseases, such as rheumatoid arthritis, Hashimoto's thyroiditis, systemic lupus erythematosus, myasthenia gravis, and pernicious anemia, should also be recorded.

### Disease recurrence/onset assessment

Disease recurrence/onset defined in the protocol refers to the occurrence of new or deteriorating neurological symptoms attributable to NMOSD. Symptoms must persist for more than 24 hours and exclude other clinical factors (e.g., fever, infection, injury, mood changes, adverse drug reactions). Neurological symptoms that appear with an interval over 31 days are considered a relapse in this protocol (i.e., if the onset days of two relapses in no more than 30 days, only one relapse will be counted), and the starting date of the recurrence used in the analysis is the first relapse. New-onset neurological symptoms need to meet any of the following:

1. an increase of more than 1.0 on the EDSS;
2. an increase of at least 2.0 on one appropriate symptom-specific functional-system score for the pyramidal system, cerebellar system, brain stem, sensory system, bowel or bladder, or a single eye;
3. an increase of at least 1.0 on more than one symptom-specific functional-system score;
4. an increase of at least 1.0 on a symptom-specific functional-system score in a single eye.

A more severe NMOSD recurrence/onset often result in hospitalization, while a less severe relapses/onsets do not. Therefore, this study uses hospitalization frequency to assess whether CT103A is effective in reducing NMO-related hospitalizations. For relapses/onsets of disease, the time of onset, the number of relapses, and hospitalizations associated with the onset should be recorded.

### Imaging assessment

In this study, MRI imaging is used to evaluate NMOSD lesions, and MRI scan and enhancement of the head, cervical spine, thoracic spine, lumbar spine, and optic nerve are required. The total number of enhanced lesions or new T2 hyperintensity should be recorded.

### Functional status assessment

In this study, EDSS score is used to evaluate the status of the subjects. Compared with the baseline, once EDSS score increases, it should be regarded as deterioration (if below 5 points, increases of 1 point counts; if 5 points and above, increases of 0.5 point counts), and the deterioration rate will be calculated.

mRS is used to assess the subject's disability status and independent living ability (0 points for asymptomatic, 6 points for death).

### Visual function assessment

The bilateral latency time in VEP needs to be recorded. If the bilateral latency time are both more than 120 ms or the bilateral latency time difference is 7 ms, it is abnormal.

Visual acuity will be checked with an eye chart, and the same vision test method should be used for all study visits for each subject.

### Quality of life assessment

#### SF-36 Scale Assessment

SF-36 involves a total of 36 questions and is a multifunctional health survey.

#### Changes in the quality of life scale EQ-5D

EQ-5D scale assesses subjects' health-related quality of life across 5 dimensions (mobility, self-care, activities of daily living, pain/discomfort, and anxiety/depression).

#### VAS pain assessment

VAS can be used to assess the level of pain experienced by a subject.

#### FACIT-fatigue assessment

FACIT-fatigue scale is a 13-item measure that assesses self-reported fatigue and its impact upon daily activities and function. .

### Other exploratory research blood sample collection and testing

Table 13 CAR-T-related serum inflammatory factors

| Ferritin | C reactive protein |
| --- | --- |
| IL-6 | procalcitonin |

### Exploratory study blood sample processing

Peripheral blood collection and processing:

All blood samples in the experiment are collected as whole blood according to the scheduled time points; blood samples are collected into pre-labeled anticoagulated blood collection tubes, which should be immediately turned up and down several times to mix thoroughly. If it cannot be processed immediately, the sample tube should be placed in a refrigerator at 2~8°C, but no more than 24 hours.

Blood samples should be transported in accordance with laboratory regulations.

# Biological sample analysis

The biological sample analysis test related to the pharmacokinetic study shall be in accordance with the basic requirements of biological sample analysis methods in the "Guidelines for the Validation of Quantitative Analysis of Biological Samples" and the relevant regulations in the "Guidelines for the Management of Biological Sample Analysis Laboratories for Drug Clinical Trials (Trial)". And it shall also be in accordance with the company/laboratory's requirements for biological sample analysis experimental plan and SOP to conduct qualitative and quantitative analysis of blood samples.

Analysis of peripheral blood CAR-T cell detection and biological samples related to lentivirus copy number research shall be carried out in accordance with the relevant requirements of the testing company/laboratory.

# Statistical Analysis

## General principles

Statistical analysis will be calculated using statistical analysis software SAS 9.4 or above. Since there is no hypothesis test in this study, descriptive statistical analysis methods are mainly used for statistical analysis in this study. Measurement data will be described as the number of cases, mean, standard deviation, quartile, minimum value and maximum value. Count data will be described as the frequency (composition ratio) in statistical description.

The software Phoenix WinNonlin 8.0 or higher (Pharsight Corp., Mountain View, CA, USA) is intended to be used for pharmacokinetic analysis, to calculate PK parameters for the copy number and concentration of BCMA CAR-T amplified in peripheral blood, and to compare the main pharmacokinetic parameters of each dose group. Descriptive statistics are presented by using the number of cases, mean, standard deviation, coefficient of variation, median, minimum, maximum, geometric mean and geometric mean coefficient of variation.

All missing data (including missing data due to subject dropout or early withdrawal) will not be filled, and raw data will be presented in the form of a list for unused data and illogical data.

Details of statistical analysis will be described in a separate statistical analysis plan. The statistical analysis plan will be finalized before the database is locked. Statistical analysis will be carried out in strict accordance with the finalized statistical analysis plan; if there are new statistical analysis requirements after finalization, new statistical analysis content will be added to the finalized version of the statistical analysis plan. but finalized statistical analysis content will not be modified before the library is locked.

## Population analysis

The analysis population of this study includes screening subject data set, enrollment analysis set, lymphodepletion analysis set, DLT evaluable analysis set, safety analysis set, full analysis set, repeated treatment analysis set (Efficacy Set), pharmacokinetic analysis set and pharmacodynamic analysis set.

The main statistical analysis sets are as follows:

- Screening Analysis Set (ENS, All Subjects Enrolled Set): All subjects who sign informed consent;
- Enrollment Analysis Set (EAS): Subjects who receive PBMC collection in ENS;
- Lymphodepletion Analysis Set: Subjects who receive lymphodepletion in EAS;
- DLT evaluable Analysis Set: Subjects who complete sufficient lymphodepletion chemotherapy and complete safety assessment within 28 days after CT103A cell infusion, or subjects with observed DLT;
- Safety Analysis Set (SS, Safety Set): All subjects who receive cell infusion and has at least one follow-up safety visit. SS will be mainly used for safety analysis;
- Full Analysis Set (FAS, Full Analysis Set): All enrolled subjects who receive CT103A cell infusion and undergo post-infusion observation;
- Repeat treatment analysis set (RTAS, Re-treatment Analysis Set): All enrolled subjects who receive CT103A cell reinfusion and repeated treatment;
- Pharmacokinetic analysis set (PKS, PK set): All enrolled subjects who receive CT103A cell infusion and have at least one post-dose PK data document, without events that significantly affect PK results;
- Pharmacodynamic analysis set (PDS, PD set): All enrolled subjects who receive cell infusion and have at least one post-dose PD data document, without events that significantly affect the pharmacodynamic data.

## Content analysis

### Case distribution

The number of cases (percentage) is used to describe the enrollment and completion of the subjects. Describe the distribution of cases in each dataset. List the reasons for early withdrawal from the trial and the medication of dropped and excluded cases.

### Protocol deviation

The protocol deviations and their severity are classified and summarized. A list of protocol deviation subjects is listed.

### Demographic data and baseline analysis

Descriptive statistical methods will be used to summarize subjects' demographic characteristics (e.g., age, gender, height, weight, etc.) and baseline characteristics (e.g., general information such as medical history).

### Treatment compliance and drug exposure analysis

Describe the information of the subject's cell infusion time and whether all the infusion has been completed by group.

### Concomitant medication and non-drug concomitant therapy

Medical coding is carried out for concomitant medications and non-drug concomitant treatments. Summarize them according to the coded system names and standard names, and listi the drug names, reasons for use, usage and dosage, and time of use of the concomitant medications.

## Safety Analysis

### AE analysis

All AEs will be classified according to the Medical Dictionary for Regulatory Activities (MedDRA) codes. All AEs, AEs that occurred during treatment (TEAEs), SAEs, TEAEs related to study drug, SAEs related to study drug, TEAEs leading to discontinuation of study medication, and AEs leading to withdrawal from the study, will be assessed by organ system, preferred term, and group to summarize the number of cases, occurrences, and incidence. In addition, the severity of AEs will also be summarized by organ system, preferred term, and group. All AEs will be listed in the form of a list;

Calculate number and percentage of CRS and neurotoxicity by group and severity.

The number and percentage of DLT in the first cycle of the dose escalation trial phase are calculated by group.

### Laboratory test

Descriptive analysis is performed on the measured values of laboratory tests and the changes from baseline after treatment by group and visit, and the changes in clinical significance judgment results before and after treatment are analyzed by cross-tabulation. At the same time, a checklist is used to describe the abnormal values of laboratory tests.

### Vital signs

Descriptive analysis of vital sign measurements and changes from baseline after treatment is carried out by treatment group and visit. The changes in clinical significance judgment results before and after treatment are analyzed by cross-tabulation. At the same time, a list is used to describe abnormal values of vital signs.

### Physical examination

The changes of clinical significance judgment results before and after treatment are analyzed in the form of cross-tabulation by treatment group and visit. At the same time, a list is used to describe the abnormal conditions of the physical examination.

## Efficacy analysis

Descriptive statistics are used to analyze the measured values of serum AQP4 antibody titers in NMOSD patients before and after treatment, and their changes from baseline.

## Pharmacokinetic (PK) Analysis

PK parameters are calculated for each subject.

- Blood drug concentration-time data analysis: Draw BCMA CAR-T cell concentration-time curve and VCN-time curve in peripheral blood; BCMA CAR-T concentration and VCN value in peripheral blood at each time point will be listed in a form of list.
- PK parameter analysis: Calculate arithmetic mean, standard deviation, coefficient of variation, median, maximum, minimum, geometric mean, and geometric mean coefficient of variation of BCMA CAR-T cell in each dose group.

## Exploratory Analysis

- - Onset assessment:
- Time from treatment beginning to first protocol-defined disease onset is calculated by treatment group, and the median time and its 95% CI are described using the Kaplan-Meier method.
- Calculate ARR and its change from baseline by treatment group.
  - By treatment group, EDSS score, the visual analog scale VAS for pain, the SF-36 scale, FACIT-fatigue scale, the quality of life scale EQ-5D and the mRS and their changes from the baseline are calculated at each visit using descriptive statistics.
  - Count number of active MRI lesions in subjects by treatment group and visit: the total number of enhanced lesions or newly added T2 hyperintensity.
  - Calculate the annualized hospitalization frequency of subjects by treatment group.
- VEP and visual acuity are analyzed by treatment group.
- Descriptive statistics are used to analyze the content of free BCMA in peripheral blood and its changes from baseline at each time point before and after treatment by each treatment group.
- Descriptive statistics are used to analyze CAR-T-related serum cytokines (such as transferrin, CRP, IL-6, etc.), human anti-CAR antibody levels, replicating lentivirus concentrations and lymphocyte subsets in each treatment group at each time point.

# Ethics and Informed Consent

## Laws and Regulations

The conduct of this trial complies with the requirements of China's "Good Clinical Practice for Drug Clinical Trials" and the "Declaration of Helsinki" (2013 edition). As in routine medical practice, investigators have the responsibility for diagnosing and treating subjects in this trial.

If any urgent safety issue occurs during the trial, measures need to be taken to avoid harm to the subject. Or if any serious violation against the trial protocol, GCP or the Declaration of Helsinki occurs, the investigator is responsible for immediately notifying the sponsor.

## Ethics Committee

Before the start of the trial, the investigator shall submit the trial protocol and the revised version of the trial protocol, the informed consent form and its update, the investigator's handbook, recruitment information (if any), and the certification documents of the investigator's qualifications to the ethics committee for approval. Any revisions to the trial protocol must be approved once again by the ethics committee.

## Informed consent

Investigators should fully explain to each subject about the nature, purpose, relevant procedures, expected time, potential risks and benefits, and any discomfort that may appear during the trial in the informed consent. Each subject must be known that he/she is voluntary to participate in the trial and that he/she may withdraw from the trial and withdraw informed consent at any time without affecting his/her subsequent treatment or relationship with the treating physician. Informed consent should be given in a standard written format and in plain language as far as possible. Each ICF must include all of the above and include a voluntary statement. Informed consent must be submitted to the ethics committee for approval. After explaining the basic content of the trial, and after the investigator has made sure that each subject who will participate in the trial understands the details of the clinical trial, each enrolled subject or his/her guardian should be asked to sign and date the ICF. If the subject is incapable of reading, an impartial witness should witness the entire informed consent process, and the witness should sign and date the ICF, too. Subjects should read and consider their statements before signing and dating them, and should obtain a copy of the ICF after signing. Subjects are not allowed to enter the trial without obtaining informed consent and signing the ICF.

# Clinical trial data management and storage

## Requirement of investigator to fill in data

- 1. For each subject who signs ICF, his/her study information should be recorded in source files carefully and in detail, with no blank items or missing items (a blank space and a horizontal line are not allowed).
  2. All data of the source file must be checked.

For data that is significantly higher or outside the clinical acceptable range, it must be verified and explained necessarily by the investigators.

## EDC data management

- 1. Electronic Case Report Form (eCRF): The data administrator builds eCRF according to the experimental protocol design, and sets up the logical verification according to the logical verification plan (DVP), which is released for use after passing the test and being approved by the sponsor.
  2. Data entry: The EDC data comes from the original records, and the data entry personnel will fill the subject visit data into the EDC in time according to the EDC instructions.
  3. On-site verification of source data (SDV): The inspector checks the consistency between the EDC data and the source data, and questions can be raised if there is any problem.
  4. Data questions and answers: Questions include system questions of EDC logic verification, and manual questions from monitors and data administrators. The investigators need to answer questions in a timely manner. Data administrators and monitors respond to answers and raise re-issue questions if necessary until the data is "clean".
  5. Investigator's signature: After the data entry is completed and SDV is passed, the investigator will conduct review and confirmation with an electronic signature. If there is any data revision after signing, it needs to be re-signed.
  6. Database lock: After the principal investigator, sponsor, statistical analyst and data administrator jointly sign the database locking record, the data administrator will lock the database.
  7. Database submission: The data administrator submits the database to the statistician.
  8. EDC archive: The EDC of each subject will be saved as a PDF electronic document.
  9. Data management report: It is written by the data administrator.
  10. EDC shutdown: After the statistical analysis is completed, the data administrator shuts down the database.

## External data transfer

If applicable, an external data transfer agreement will be signed for external data management in accordance with the DMP.

## Source file

According to the requirements of relevant regulations, investigators should properly keep the original records of clinical research. The investigator must keep the study protocol, CRF, relevant correspondence with the sponsor, original medical documents and other trial documents for 5 years after the end of the study, or wait until the sponsor notifies and allows the investigator before destroying these documents.

# References

1. Huang Jing, Jin Yuting & Gao Haizhai. Current status and progress of research on autoimmune diseases of the nervous system. Chinese Journal of Laboratory Medicine **41** , 917–921 (2018).

2. Xu Yan & Wang Weizhi. Interpretation of the 2015 new diagnostic criteria for neuromyelitis optica spectrum disorders. Chinese Journal of Neurology **49** , 499–501 (2016).

3. Neuroimmunology Branch of Chinese Society of Immunology, Neuroimmunology Group of Neurology Branch of Chinese Medical Association & Neuroimmunology Professional Committee of Neurology Branch of Chinese Medical Doctor Association. Guidelines for the diagnosis and treatment of neuromyelitis optica spectrum disorders in China. Neuroimmunology and Neurology of China Journal **23** , 155–166 (2016).

4. Wei Shihui, Yang Mo & Wu Weiping. Epidemiological study of neuromyelitis optica spectrum disorders. Chinese Journal of Ophthalmology **55** , 234–240 (2019).

5. Sadelain, M. Chimeric Antigen Receptors: A Paradigm Shift in Immunotherapy. *Annual Review of Cancer Biology* **1** , 447–466 (2017).

6. Jensen, MC & Riddell, SR Designing chimeric antigen receptors to effectively and safely target tumors. *Current Opinion in Immunology* **33** , 9–15 (2015).

7. June, CH & Sadelain, M. Chimeric Antigen Receptor Therapy. *New England Journal of Medicine* **379** , 64–73 (2018).

8. Rickert, RC, Jellusova, J. & Miletic, AV Signaling by the tumor necrosis factor receptor superfamily in B-cell biology and disease. *Immunol. Rev.* **244** , 115–133 (2011).

9. Moisini, I. & Davidson, A. BAFF: a local and systemic target in autoimmune diseases. *Clin. Exp. Immunol.* **158** , 155–163 (2009).

10. Novak, AJ *et al.* Expression of BCMA, TACI, and BAFF-R in multiple myeloma: a mechanism for growth and survival. *Blood* **103** , 689–694 (2004).

11. Dalakas, MC Invited article: inhibition of B cell functions: implications for neurology. *Neurology* **70** , 2252–2260 (2008).

12. Beecher, G., Putko, BN, Wagner, AN & Siddiqi, ZA Therapies Directed Against B-Cells and Downstream Effectors in Generalized Autoimmune Myasthenia Gravis: Current Status. *Drugs* **79** , 353–364 (2019).

13. López De Padilla, CM *et al.* BAFF expression correlates with idiopathic inflammatory myopathy disease activity measures and autoantibodies. *J. Rheumatol.* **40** , 294–302 (2013).

14. Coquery, CM & Erickson, LD Regulatory roles of the tumor necrosis factor receptor BCMA. *Crit. Rev. Immunol.* **32** , 287–305 (2012).

15. Papadopoulos, MC, Bennett, JL & Verkman, AS Treatment of neuromyelitis optica: state-of-the-art and emerging therapies. *Nat Rev Neurol* **10** , 493–506 (2014).

16. Ali, SA *et al.* T cells expressing an anti-B-cell-maturation-antigen chimeric antigen receptor cause remissions of multiple myeloma. *Blood* blood-2016-04-711903 (2016) doi:10.1182/blood-2016-04 -711903.

17. Cohen, AD *et al.* B-Cell Maturation Antigen (BCMA)-Specific Chimeric Antigen Receptor T Cells (CART-BCMA) for Multiple Myeloma (MM): Initial Safety and Efficacy from a Phase I Study. *Blood* **128** , 1147–1147 (2016).

18. Raje, NS *et al.* bb2121 anti-BCMA CAR T-cell therapy in patients with relapsed/refractory multiple myeloma: Updated results from a multicenter phase I study. *JCO* **36** , 8007–8007 (2018).

19. Fan, F. (Xiaohu) *et al.* Durable remissions with BCMA-specific chimeric antigen receptor (CAR)-modified T cells in patients with refractory/relapsed multiple myeloma. *JCO* **35** , LBA3001–LBA3001 (2017).

20. Wingerchuk, DM *et al.* International consensus diagnostic criteria for neuromyelitis optical spectrum disorders. *Neurology* **85** , 177–189 (2015).

21. Lee, DW *et al.* ASTCT Consensus Grading for Cytokine Release Syndrome and Neurologic Toxicity Associated with Immune Effector Cells. *Biol. Blood Marrow Transplant.* **25** , 625–638 (2019).
